# Integrating a genome‐wide association study with a large‐scale transcriptome analysis to predict genetic regions influencing the glycaemic index and texture in rice

**DOI:** 10.1111/pbi.13051

**Published:** 2019-01-24

**Authors:** Roslen Anacleto, Saurabh Badoni, Sabiha Parween, Vito M. Butardo, Gopal Misra, Rosa Paula Cuevas, Markus Kuhlmann, Trinidad P. Trinidad, Aida C. Mallillin, Cecilia Acuin, Anthony R. Bird, Matthew K. Morell, Nese Sreenivasulu

**Affiliations:** ^1^ International Rice Research Institute Los Baños Philippines; ^2^ Department of Chemistry and Biotechnology Faculty of Science, Engineering and Technology Swinburne University of Technology Hawthorn Vic. Australia; ^3^ The Leibniz Institute of Plant Genetics and Crop Plant Research (IPK) Gatersleben Germany; ^4^ Food and Nutrition Research Institute (FNRI) Taguig City Philippines; ^5^ CSIRO Health and Biosecurity Adelaide SA Australia

**Keywords:** glycaemic index, final viscosity, genome‐wide association study, transcriptome‐wide association study, gene‐regulatory network analysis, whole‐genome bisulfite sequencing

## Abstract

Reliably generating rice varieties with low glycaemic index (GI) is an important nutritional intervention given the high rates of Type II diabetes incidences in Asia where rice is staple diet. We integrated a genome‐wide association study (GWAS) with a transcriptome‐wide association study (TWAS) to determine the genetic basis of the GI in rice. GWAS utilized 305 re‐sequenced diverse *indica* panel comprising ~2.4 million single nucleotide polymorphisms (SNPs) enriched in genic regions. A novel association signal was detected at a synonymous SNP in exon 2 of LOC_Os05g03600 for intermediate‐to‐high GI phenotypic variation. Another major hotspot region was predicted for contributing intermediate‐to‐high GI variation, involves 26 genes on chromosome 6 (GI6.1). These set of genes included *GBSSI*, two hydrolase genes, genes involved in signalling and chromatin modification. The TWAS and methylome sequencing data revealed *cis*‐acting functionally relevant genetic variants with differential methylation patterns in the hot spot GI6.1 region, narrowing the target to 13 genes. Conversely, the promoter region of *GBSSI* and its alternative splicing allele (G allele of *Wx* ^*a*^) explained the intermediate‐to‐high GI variation. A SNP (C˃T) at exon‐10 was also highlighted in the preceding analyses to influence final viscosity (FV)*,* which is independent of amylose content/GI **.** The low GI line with GC haplotype confirmed soft texture, while other two low GI lines with GT haplotype were characterized as hard and cohesive. The low GI lines were further confirmed through clinical *in vivo* studies. Gene regulatory network analysis highlighted the role of the non‐starch polysaccharide pathway in lowering GI.

## Introduction

The global rise in obesity has affected 2.1 billion adults and 422 million of these adults have Type II diabetics; thus, obesity is the leading cause of non‐communicable disease (NCD) (Brand‐Miller, 2003; Scully, 2012; Shetty, 2012; World Health Organization, 2016). Various factors, such as urbanization, the shift to an energy‐rich Western diet, a sedentary lifestyle and genetic predisposition, impact the development of diabetes mellitus. The glycaemic index (GI) is a measure of the effect of food on blood glucose levels upon digestion. The rapid digestion of starch can cause a sharp increase in blood glucose levels that is detrimental to those suffering from Type II diabetes (Scully, 2012; Shetty, 2012). Slowing starch digestibility by converting rice to a source of healthy carbohydrates is an important nutritional intervention among rice consuming populations. A low‐GI diet including slower digestible rice improves metabolic health and is helpful in improving insulin sensitivity, which can mitigate the prevalence of associated diseases (Brand‐Miller, 2003).

White or polished rice (*Oryza sativa* L.) is a staple food for most people in Asia and half of the world's population. Despite the nutritional advantages of brown rice, >99% of the rice consumed is white or polished rice. The major storage product of polished rice grain is starch. Waxy to intermediate amylose rice is mostly composed of easily digestible starch which has approximately 6%–8% protein. The consumption of such rice is associated with a sudden increase in blood glucose levels. Hence, alterations in the starch structure to lower digestibility can provide a slow and steady supply of energy while significantly lowering the GI (Butardo *et al*., 2011, 2017; de Guzman *et al*., 2017). Generally, brown rice has an intermediate GI (Atkinson *et al*., 2008) that increases when polished to white rice (Karupaiah *et al*., 2011). The GI of polished rice from different accessions varies from 48 to 92 (Atkinson *et al*., 2008; Fitzgerald *et al*., 2011), suggesting that sufficient genetic diversity exists in the gene pool for this phenotype. However, minimal progress has been achieved thus far in defining the genetic basis of GI due to the lack of robust, cost‐effective high‐throughput phenotyping platforms for screening a diverse germplasm panel.

The texture of cooked rice is an extremely important attribute that determines the consumer acceptance of rice varieties in different regions of the world. Cooked rice texture is governed by multiple factors including the composition of per cent of amylose and amylopectin (Li *et al*., 2016; Ong and Blanshard, 1995). Though many rice varieties possess 90% starch in the grain, they differ in amylose‐to‐amylopectin ratios and thus differ in digestibility and cooked rice textural properties (Frei *et al*., 2003; Hu *et al*., 2004; Rani and Bhattachrya, 1995). Moreover, the genomic variations lying in the genic region of *waxy* (*GBSSI*) locus influence the cooking quality properties was preferentially selected during the course of domestication and breeding programs in rice (Chen *et al*., 2008; Larkin and Park, 2003).

A number of approaches including mutagenesis, genetic engineering and genome editing have been adopted in previous studies investigating the starch biosynthesis pathway to produce high‐amylose rice with a low GI attribute (Butardo *et al*., 2011, 2012; Fitzgerald *et al*., 2011; Nishi *et al*., 2001; Sun *et al*., 2017; Yano *et al*., 1985; Zhu *et al*., 2012). In particular, mutations in starch synthase IIa and IIb (*SSIIa, SSIIb*), soluble starch synthase IIIa (*SSIIIa*) and starch branching enzymes (*SBEs*) were targeted in the background of *Wx* ^*a*^ allele to generate high amylose lines (Wei *et al*., 2010; Yang *et al*., 2012; Zhou *et al*., 2016). However, high amylose rice has undesirable cooking and eating qualities (Butardo *et al*., 2017). Studies involving low‐GI in rice that maintains the considerable organoleptic attributes are still elusive. Additionally, a rich gene pool that not only sufficiently covers the GI range but also combines good eating and cooking qualities can facilitate the breeding of high‐quality healthier rice that is preferred by health‐conscious consumers (Wang *et al*., 2007). These efforts might eventually facilitate the combination of favourable alleles for low‐GI attributes with good organoleptic properties matching preferred consumer preferences.

To understand the genetic basis of GI, this study used 2 419 731 SNP markers from 305 *indica* accessions and performed GWAS on *in vitro* GI values. eQTL analysis was subsequently done through TWAS to narrow down the GWAS‐implicated genes as a fine‐mapping strategy to come up with candidate genes. A similar approach was taken on amylose content and final viscosity to determine the common genes acting on both the GI and the cooking and eating qualities of rice. Accessions found to have low GI were validated through *in vivo* human clinical studies. Independent measurements of cooked rice texture were obtained through the texture analyzer and through a descriptive human sensory panel.

## Results

### Delineating relationship between predictive glycaemic index response and cooking quality in diversity panel of indica

**Figure 1:**
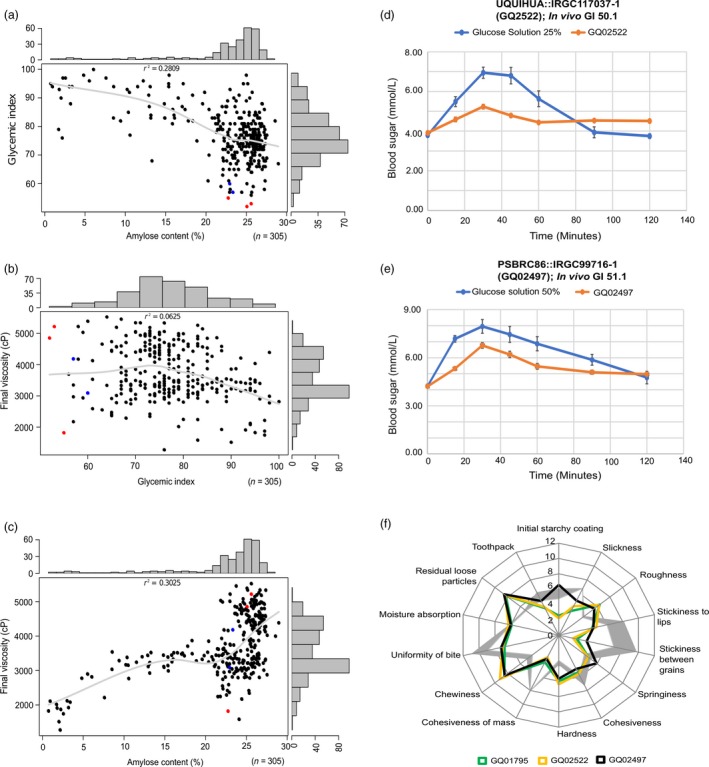
Correlation among *in vitro* glycemic index (GI), eating (amylose content; AC) and cooking (final viscosity; FV) qualities of 305 rice germplasm lines. The scatter plots showed (a) the weak correlation of AC with GI (Pearson's *r* ^2^ = 0.2809), (b) practically no meaningful relationship between GI and FV and (c) correlation between AC and FV with *r* ^2^ = 0.30. Genotypes that had low GI values (GI ≤ 55) were shown as red dots whereas lines with GI value between 56 and 60 (intermediate GI) were randomly selected and plotted as blue dots. All three data sets (GI, AC and FV) were transformed into a distribution with normally distributed residuals that had mean 0 and unit variance prior to genetic analysis without regard to their raw data distribution. (d and e) represent the plots for *in vivo* GI evaluation of two landraces GQ02522 and GQ02497 identified to be low GI based on cohort studies of at least 10 human subjects with reference to control (glucose solution, blue line). (f) Overlaid sensory and texture profiling of three low GI lines using 14 textural attributes representing the characteristic variations in the radar graph. Fourteen attributes in case of the three accessions were evaluated in comparison to ‘Dinorado’ a popular rice variety in the Philippines (depicted by grey areas, wideness of which corresponds to the differences between the profiles generated in the two sessions of sensory test).

We selected a diverse panel of 305 *indica* rice landraces covering diverse amylose range from 0% to 30% (Figure 1a,c) from 51 countries with existing whole genome sequence data (The 3000 Rice Genomes Project, 2014), and conducted *in vitro* GI phenotyping using the starch hydrolysis index (HI) method (Figure S1). We observed high phenotypic diversity in the GI ranging from low (52–55) to high (~100). Three out of the 305 lines tested were found to have low digestibility based on the preliminary HI screening. The amylose content (AC) was weakly inversely correlated with the GI, with a low coefficient of determination (*r* ^2 ^= 0.28) (Figure 1a). Additionally, the GI and final viscosity (FV), which is a proxy measure of eating quality, were found to be unrelated (*r* ^2 ^= 0.06) (Figure 1b). This finding was also evidenced in three cultivars, which despite bearing the lowest GI values plotted at opposite ends of the FV range (Figure 1b). Likewise, in the case of AC versus FV, a low positive correlation (*r* ^2 ^= 0.30) was observed (Figure 1c).

### Validating low glycaemic effect of rice accessions using clinical GI studies

Of the three low digestibility accessions, we selected two low GI accessions with differential texture (GQ2522 and GQ02497) for clinical validation on human volunteers in a cohort study initially involving 13 human subjects (Figure 1d‐e, Table S1). We screened subjects using Body Mass Index (BMI), Fasting Blood Sugar (FBS) and Haemoglobin A1C to eliminate high variability subjects. We eventually selected 12 panel members for studying *in vivo* GI responses due to their more homogenous physiological response. Our clinical validation satisfied well above the minimum of 10 human volunteers required for clinical testing for GI based on ISO 26642:^2010^. The mean GI values obtained after consumption were compared with that of a reference glucose standard of comparable carbohydrate equivalence. The *in vivo* GI response in the employed subjects were assessed with the mean GI value of 50.1 for GQ2522 (Figure 1d, Table S1), while for GQ02497 mean GI value was 51.1 (Figure 1e, Table S1). These results were consistent with their corresponding *in vitro* mean GI values. Hence, the employed approach of screening large germplasm (*n* = 305) through *in vitro* digestibility method proved to be useful, prior to subjecting selected accessions (*n* = 2) for a more rigorous clinical GI validation.

### Identifying rare rice germplasm with low glycaemic index and superior sensory attributes

Because most low GI varieties in the market today have hard texture, we deliberately looked for a unique phenotypic combination of low GI and soft texture, if there is any that can be found in the germplasm collection. Such rare accessions in the gene pool can potentially be important allele donors for developing rice varieties with a good balance between low GI and good eating quality. Because rice samples were analysed by sensory panelists over the span of 2 days, it was important to test if the panelists provided consistent results on both days and thus, Dinorado a popular variety widely preferred in the Philippines was used as internal benchmark quality control to assess textural attributes. The averages of each textural attribute of Dinorado (from the two evaluations) were used to generate radar graphs with grey shading (Figure 1f). Results indicate that there was no significant difference in ratings provided by the panelists across 14 textural attributes during these two rice tasting sessions using Dinorado as bench mark variety (Table S2).

By comparing three low‐GI milled seed samples with the soft quality benchmark Dinorado, panelists were able to distinguish all three low‐GI lines for unique textural properties such as residual loose particles, chewiness, roughness and hardness (Figure 1f). GQ02522, GQ01795 represented similar kind of sensory profile and were perceived to be rougher, harder and chewier and possessing more residual loose particles than Dinorado (Tables S3 and S4). GQ02522 was found to be harder than GQ02497, while GQ02497 was characterized as more adhesive and cohesive than GQ02522 (Tables S3 and S5). The panel‐based texture data attributes were verified by quantification using instrument‐based texture profile analyser (TPA) (Table S6). Conversely, GQ02497 was perceived to have a similar initial starchy coating, roughness and hardness as with Dinorado differing it with the rest of the two low GI lines. Nevertheless, GQ02497 was found to be less sticky between grains and to have less cohesiveness of mass. Notably, the low‐GI sample, GQ02497 was perceived to have similar initial starchy coating, as with Dinorado (Table S5). Increase in the perception of initial starchy coating (perceived in case of GQ02497) is one of the crucial factors for populations who prefer Dinorado type rice.

### Application of genome‐wide association study to identify the genetic regions that influence glycaemic index and proxy tests of textural properties in rice

**Figure 2:**
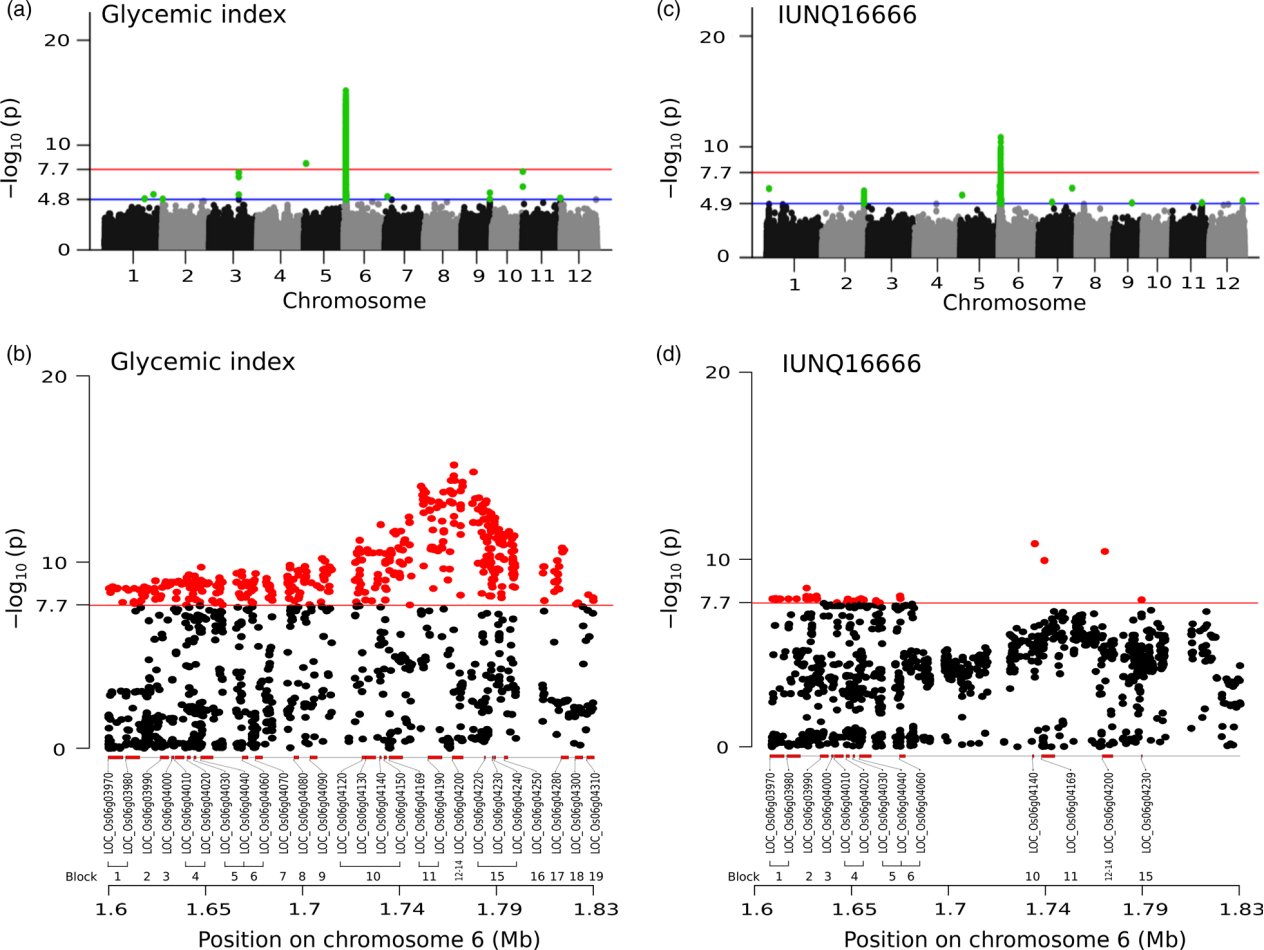
Genome‐wide association study of glycemic index and transcription‐wide association study (TWAS) highlighting the expression level of *GBSS I*. (a) Manhattan plot shows significant association signals in chromosomes 5 and 6 indicated by the red line at –log(*p*) = 7.7 that represents the Bonferroni‐corrected *P*‐value significance threshold. The blue line at –log(*p*) = 4.8 is the suggestive line represented by the association *P*‐value that corresponds to the *q*‐value<0.5 (false discovery rate). A C→T synonymous SNP (1 525 361 bp) in exon 2 of LOC_Os05g03600 (encodes a signalling receptor kinase) is a significant SNP in chromosome 5. In chromosome 6, we found a 230 kb region (1.60–1.83 Mb) that we refer to in this manuscript as GI6.1. (b) Zoomed‐in GI6.1 region, where multiple significant SNPs have implicated within a total of 26 genes that have been validated in the gene‐set analysis, distributed in 19 different linkage disequilibrium (LD) blocks. (c) Manhattan plot of the transcription‐wide association study that highlighted the expression level of *GBSS I*. The red and blue lines were as described in (a) with the exception of the suggestive line being a little bit higher at 4.9 due mainly to the adjustment made for false positives. (d) A plot of TWAS with –log10 *P*‐values in the similar GI6.1 region, based on the expression QTL (eQTL) analysis, narrowing down to 13 candidates genes in the GI6.1 region.

**Figure 3:**
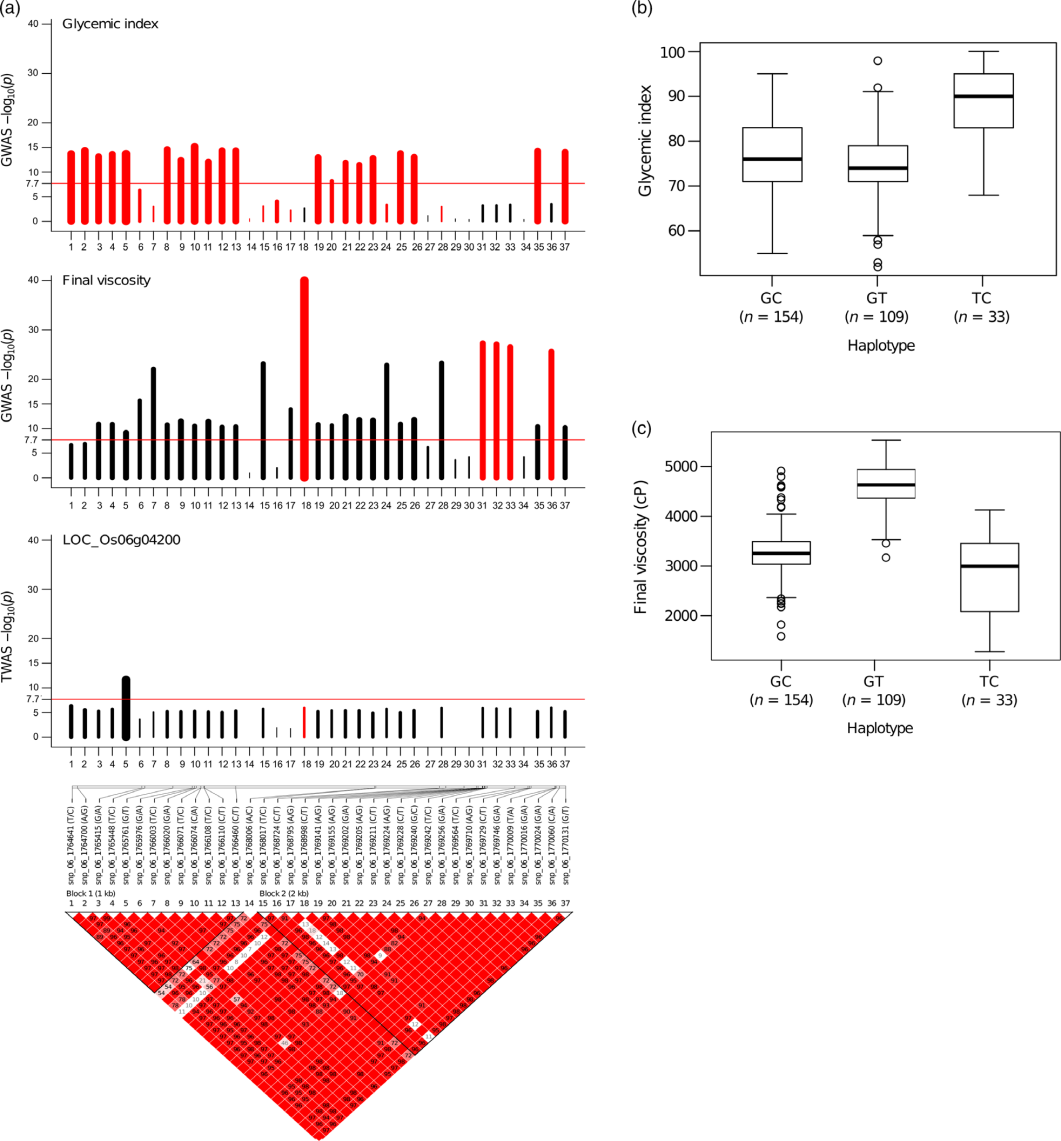
Summary for genomic variation present within the gene and 1 kb promoter region of *granule‐bound starch synthase I* (*GBSS I*). (a) The –log plot of the association *P*‐values for the glycemic index (GI), shown in the top graph. A red bar indicates that the negative effect of the allele on the trait, while a black bar indicates a positive effect. Bar thickness reflects the relative effect size of the respective allele, as beta value. The middle one is the –log plot of the association *P*‐values for final viscosity (FV). The third track is the –log plot of the *P*‐values of the *cis*‐eQTL analysis done on the expression of *GBSS I* using TWAS. The bottom track that shows SNP IDs with their alleles, the placement of the SNPs on the chromosome segment, and the haplotype blocks calculated using Gabriel's algorithm implemented in Haploview 4.2. The two linkage blocks within the gene were formed from the linkage break at the A→C SNP (located at chromosomal position 1 768 006 bp) in *GBSS I*. This SNP is situated two base pairs away from the 3′ end of exon 6. (b and c) explained the haplotypes lying within *GBSS I* and contributing influence the GI and FV values, respectively. The replacement of T with G allele of the splice junction SNP of intron1 predisposes with converting high to intermediate GI, whereas a conversion of T to C allele in the exon 10 SNP correlated to shifting FV from high to intermediate.

We performed an efficient mixed model association expedited (EMMAX) (Kang *et al*., 2010) test in this study to determine the association between the GI trait and 2 419 731 high‐quality single nucleotide polymorphism (SNP) markers mapped to the Nipponbare reference genome (release 7) that were specifically designed to cover up to 3.7 kb both upstream and downstream of every gene (Figure 2a,b). We used log(*p*)>7.67 (P_GWAS_) as the genome‐wide significance threshold after a Bonferroni correction. Subsequently, gene and gene‐set analyses were conducted using magma (de Leeuw *et al*., 2015) on GWAS summary data to further narrow down the candidates. We found a synonymous T→C SNP (snp_05_1525361, −log(*p*) = 8.24) in LOC_Os05g03600 (encodes a signalling receptor kinase) on chromosome 5 with an positive allelic effect (β = 0.83) (Table S7), where altered allele (C) leads to an increase in the GI value. Furthermore, a 230 kb (1.60–1.83 Mb) hotspot region on chromosome 6 (designated GI6.1) encompassing 26 significant genes accounted for 88.7% of the total variation in GI (Figure 2b). The genome‐wide linkage disequilibrium (LD) decay, which was calculated using equally spaced SNPs, showed that *r* ^2^ decreased to 0.24 at ~163 kb (Figure S2a,b). Interestingly, the rate of the LD decay was faster in GI6.1, and the *r* ^2^ decreased to 0.16 at ~60 kb, which explains the number of linkage blocks in this hotspot region that represents a very high recombination rate. Furthermore, calculating the long‐range LD within this region revealed a hump 400–650 kb before a monotonic drop. The LD calculation identified 19 linkage blocks within GI6.1 (Table 1 and Figure 2b). Phylogenetic analysis conducted based on groups constructed from the significant SNPs identified from GI6.1 LD‐block 1–6 and block 15, suggests a cleaner separation of high versus intermediate/low GI phenotypes (Figures 3a,b and S3). Three LD blocks co‐localized within the granule‐bound starch synthase I (*GBSSI* or *Waxy* gene) locus (LOC_Os06g04200) including its 1 kb promoter region (Figures 3a and S4a,b). However, the GBSS region identified in block 12 to 14 showed moderate GI range distinction (Figure S3).

**Table 1:** Genes associated with glycaemic index identified by genome‐wide association study and gene set analysis

Gene ID	Chr	Block	Start	End	Strand	nSNPs	*n*	*z*‐stat	*P* value	Bonferroni	FDR	Annotation
LOC_Os05g03600	5	1	1 523 847	1 529 707	+	5	303	3.7344	9.41e‐05	2.54e‐03	1.02e‐04	Retrotransposon protein, putative, unclassified, expressed
LOC_Os06g03970^a^	6	1	1 605 460	1 612 409	+	23	303	5.1343	1.42e‐07	3.82e‐06	2.55e‐07	Receptor‐like protein kinase 5 precursor, putative, expressed
LOC_Os06g03980^a^	6	1	1 613 664	1 620 183	−	26	301	5.2933	6.01e‐08	1.62e‐06	1.25e‐07	Expressed protein
LOC_Os06g03990^a^	6	2	1 629 717	1 633 629	+	10	302	5.5093	1.80e‐08	4.86e‐07	5.78e‐08	Aminotransferase, classes I and II, domain containing protein, expressed
LOC_Os06g04000	6	3	1 635 096	1 636 029	+	1	299	5.3989	3.35e‐08	9.05e‐07	7.65e‐08	Peptidyl‐prolyl cis‐trans isomerase, putative, expressed
LOC_Os06g04010^a^	6	4	1 636 524	1 640 883	−	17	302	5.7231	5.23e‐09	1.41e‐07	2.29e‐08	GAGA‐binding protein, putative, expressed
LOC_Os06g04020^a^	6	4	1 642 214	1 643 800	−	8	302	4.8677	5.65e‐07	1.52e‐05	8.97e‐07	Histone H1, putative, expressed
LOC_Os06g04030^a^	6	5	1 645 414	1 646 326	−	5	301	4.7079	1.25e‐06	3.38e‐05	1.74e‐06	Histone H3, putative, expressed
LOC_Os06g04040^a^	6	6	1 648 595	1 654 300	+	15	302	5.4965	1.94e‐08	5.23e‐07	5.78e‐08	WD domain, G‐beta repeat domain containing protein, expressed
LOC_Os06g04060^a^	6	6	1667929	1 670 634	+	12	301	5.3964	3.40e‐08	9.18e‐07	7.65e‐08	Expressed protein
LOC_Os06g04070	6	7	1 674 045	1 677 406	−	5	298	4.0391	2.68e‐05	7.24e‐04	3.15e‐05	Pyridoxal‐dependent decarboxylase protein, putative, expressed
LOC_Os06g04080	6	8	1 691 885	1 694 140	+	9	299	5.1816	1.10e‐07	2.97e‐06	2.12e‐07	Glycosyl hydrolases family 17, putative, expressed
LOC_Os06g04090	6	9	1 699 371	1 702 859	−	7	300	4.7765	8.92e‐07	2.41e‐05	1.34e‐06	No apical meristem protein, putative, expressed
LOC_Os06g04120	6	10	1723575	1725392	+	2	302	5.0607	2.09e‐07	5.64e‐06	3.52e‐07	Hypothetical protein
LOC_Os06g04130	6	10	1 725 313	1 729 996	−	7	302	5.7013	5.94e‐09	1.61e‐07	2.29e‐08	Lung seven transmembrane domain containing protein, putative, expressed
LOC_Os06g04140^a^	6	10	1 731 813	1 732 565	−	1	298	4.7024	1.29e‐06	3.47e‐05	1.74e‐06	Expressed protein
LOC_Os06g04150	6	10	1 733 935	1 735 135	−	5	302	5.4788	2.14e‐08	5.78e‐07	5.78e‐08	Magnesium‐protoporphyrin O‐methyltransferase, putative, expressed
*LOC_Os06g04169* a	6	11	1 736 249	1 742 581	−	28	301	5.9279	1.53e‐09	4.14e‐08	1.02e‐08	Hydrolase, alpha/beta fold family domain containing protein, expressed
LOC_Os06g04190	6	11	1 754 351	1 760 884	−	26	301	5.8932	1.89e‐09	5.11e‐08	1.02e‐08	Rad1, putative, expressed
*LOC_Os06g04200* a	6	12,13,14	1 765 622	1 770 656	+	33	300	6.6434	1.53e‐11	4.14e‐10	4.14e‐10	Starch synthase, putative, expressed
LOC_Os06g04220	6	15	1 780 472	1 781 052	+	7	301	3.2284	6.22e‐04	1.68e‐02	6.22e‐04	Expressed protein
LOC_Os06g04230^a^	6	15	1 784 311	1 784 827	+	2	299	5.9086	1.73e‐09	4.66e‐08	1.02e‐08	Expressed protein
LOC_Os06g04240	6	15	1 785 159	1 785 889	−	3	301	5.9713	1.18e‐09	3.18e‐08	1.02e‐08	Expressed protein
LOC_Os06g04250	6	16	1 789 836	1 791 394	+	3	302	3.2908	4.99e‐04	1.35e‐02	5.19e‐04	Phosphate‐induced protein 1 conserved region domain containing protein, expressed
LOC_Os06g04280	6	17	1 816 175	1 819 804	+	8	300	4.4773	3.78e‐06	1.02e‐04	4.86e‐06	3‐phosphoshikimate 1‐carboxyvinyltransferase, chloroplast precursor, putative, expressed
LOC_Os06g04300	6	18	1 822 766	1 826 383	−	6	300	3.7833	7.74e‐05	2.09e‐03	8.70e‐05	tRNA 2‐phosphotransferase 1, putative, expressed
LOC_Os06g04310	6	19	1 828 169	1 831 736	−	14	298	4.2439	1.10e‐05	2.97e‐04	1.35e‐05	Expressed protein

These genes were implicated by SNPs that had genome‐wide association *P*‐values below the Bonferroni‐corrected threshold of 2.066345e‐08 (calculated as 0.05/*m*, where *m *=* *2 419 731 (SNP markers).

Chr indicates the chromosome number; Block, the linkage block number as calculated from plink2 ‐–blocks within the implicated genomic region; Start and End, the start and end 1‐based integer coordinates of the gene's location within the chromosome; Strand, defined as + (forward) or − (reverse); nSNPs, number of SNPs within the gene; *n*, number of individuals tested; *z*‐stat, a probit transformation of the gene's *P*‐value computed during the gene analysis step; *P*‐value, calculated from magma; Bonferroni and FDR, Bonferroni‐corrected and False Discovery Rate corrected *P*‐values, respectively; Annotation, gene annotation reported in Rice Genome Annotation Project Release 7.

a Genes associated with the variation in expression levels of the *Waxy* locus identified by *cis*‐eQTL analysis.

*Italic gene Ids* representing the candidate genes validated through SMR analysis.

The defined haplotype within *GBSSI* explained phenotypic variation between high and intermediate GI (Figures 3a,b, S5 and S6a,b). Two linkage blocks within the *Waxy* locus (5034 bp in length) reflected an unusual LD decay within the gene, and the first block contained a highly significant T→G splice variant at 1 765 761 bp (−log(*p*)  = 13.72, β = −0.74) lying in 5′end of intron 1 (LOC_Os06g04200.1) (Figures 3a and S4a,b). The second block contained a C→T SNP at 1 768 998 bp (in exon 10) that does not alter the GI trait but is significantly associated with FV with −log(*p*)  = 40.01, and β = −0.73 (Figures 3a, and S4b). Two‐SNP haplotype were formed based on the two highly discriminating *GBSSI* SNPs: the splice junction SNP (T→G) and the exon 10 SNP (C→T) that were particularly relevant for GI and FV, respectively (Figures 3a and S4b). The GC haplotype explained predominantly intermediate values for both GI and FV, while the GT haplotype mapped out lines with intermediate GI and high FV (Figure 3a–c). The combination of GI and cooking quality classes as explained by these two‐SNP haplotypes also tend to neatly classify lines into culturally similar regions as shown by the result of phylogenetic analysis using 636 974 SNPs (Figure S7). The cultivars originating from the Southeast Asian countries have possessed mostly GC haplotypes with softer rice texture. Conversely, germplasm across the Pacific from Mainland Asia and South American countries prominently possessed GT haplotypes. Notably, key haplotypes from India and Bangladesh form clades of GC or GT haplotypes, having *Wx* ^*a*^ (G allele), whereas, the ‐T allele explains the harder texture (Figures 3a,c, S7 and S8). In contrast, rice varieties with the TC allele were found with low‐to‐intermediate FV and high GI mainly due to the activity of the *Wx* ^*b*^ allele (Figures 3a–c and S8). Thailand, Myanmar and Laos, which are neighbouring countries in Southeast Asia, clustered in nearby clades, and most had the TC haplotype. It is interesting to note that none of the accessions in the *indica* panel had the TT haplotype (Figure S7).

Two hydrolases belonging to different linkage blocks (Figure 2a and Table 1) in GI6.1 implicated by SNPs in the exonic and regulatory regions were significantly associated with the GI variation. A non‐synonymous C→T SNP at 1 736 459 bp (‐log(*p*) = 10.64, β = −0.70) in exon 4 of LOC_Os06g04169 (encoding transmembrane glycosyl hydrolase) causes an Arg→Gln substitution (Figure S9). The T→G non‐synonymous SNP at 1 693 774 bp (‐log(*p*) = 9.57, β = −0.56) lying in LOC_Os06g04080 (encoding β‐1,3 glucan hydrolases) causes a Ser→Ala substitution. The haplotypes constructed from significant SNPs present within the candidate gene LOC_Os06g04169 showed high phenotypic variability for both GI and FV (Figure S10).

### Amylose as a covariate in the analysis of the GI and FV

To potentially identify new regions acting on GI independently of the AC, we repeated the GWAS with *in vitro* GI phenotype and added AC as a covariate using the same methods and parameters (Figure S11a,b). Although the model showed insufficient power to detect significant association signals based on the Bonferroni‐corrected threshold due to the sample size limitation, several SNPs within GI6.1 with a ‐log(*p*)>5 were identified to have putative regulatory functions. These include splice junction SNPs and those found in introns, the 5′‐UTR, 1 kb up‐ and downstream of genes, and those clustered in intergenic regions (Table S8). We found several significant intergenic SNPs in GI6.1 between LOC_Os06g04169 and LOC_Os06g04190 as well as between LOC_Os06g04195 and *GBSSI*. One SNP was found on chromosome 1 between LOC_Os01g52690 and LOC_Os01g52700. Three SNPs were found in the promoter region of *GBSSI* (Table S8).

We also performed a GWAS on the FV with AC as a covariate to identify SNPs influencing cooking quality which is independent of AC. We found highly significant association signals on chromosomes 2 (LOC_Os02g53650), chromosome 5 (intergenic between LOC_Os05g15580 and LOC_Os05g15590), chromosome 6 matching GI6.1 and chromosome 11 (intergenic between LOC_Os11g25230 and LOC_Os11g25240) (Figure S12a,b and Table S9). We found the most significant association signal at the C→T synonymous SNP (−log(*p*) = 27.09, β = −0.60) located in exon 10 of *GBSS I* (1 768 998 bp) located within GI6.1. Interestingly, this SNP strongly influences the variation in FV but is not significantly associated with GI (Figures 3c, and S12a; Tables S7 and S9).

### Identifying cis factors through expression‐trait associations (expression QTL) of glycaemic index GI 6.1 region using transcriptome‐wide association study and methylation analysis

We sub‐selected 195 rice lines from the 305 resequencing lines and generated microarray‐based gene expression data from developing grains collected at 16 days after fertilization (DAF) using a newly designed *indica* panel microarray (Agilent). TWAS was employed to identify the cis regulating elements within GI6.1 region and refining total candidates to 13 (Figure 2c,d). By performing an eQTL analysis of *GBSS1*, we identified *cis‐*regulating factors, such as its splice junction SNP (‐log(*p*) = 10.43, β = 0.83) and neighbouring genes within a 1 Mb chromosomal distance (Tables S10 and S11), including LOC_Os06g04169 (explained above). Interestingly, other *cis*‐acting genes within GI6.1 showed significant levels of co‐expression, including LOC_Os06g04010, which encodes a GAGA‐binding protein (a transcription factor family), while LOC_Os06g04020 and LOC_Os06g04030 encode the histone H1 and H3 proteins, respectively (Figure 2d and Table S10). GDSL‐motif lipase genes lying upstream of GI6.1 were also spotted. The candidates encoding the lipases include LOC_Os06g03890 and LOC_Os06g03900, identified by the A→G SNP at 1566199 bp (‐log(*p*) = 9.41, β = 0.80) and A→T SNP at 1572583 bp (−log(*p*) = 9.69, β = 0.81), respectively.

Summary‐based Mendelian randomization (SMR) analyses were performed to determine the pleiotropic associations of *cis*‐acting genetic variants to both GI (phenotype) and the expression of candidate genes came out significant after GWAS and gene set analysis (Figure 2b; Tables S10 and S11). The pleiotropic effects were distinguished from the linkage effect using a heterogeneity test (heterogeneity in dependent instruments; HEIDI) (Zhu *et al*., 2016) integrated within the SMR analysis. This linear analytical pipeline produced statistical evidence of the functional relevance of LOC_Os06g04169 (encoding transmembrane glycosyl hydrolase) and LOC_Os06g04200 (encoding GBSSI) influencing GI (Figure 2b,d). The pleiotropic SNPs implicating both genes included the C→T SNP at 1 742 115 bp (P_GWAS_ = 2.28e‐11, P_*cis*‐eQTL_ = 2.84e‐04, P_SMR_ = 1.30e‐03, P_HEIDI_ = 1.66e‐01) and the splice junction SNP in the *Waxy* locus (P_GWAS_ = 1.92e‐14, P_*cis*‐eQTL_ = 2.07e‐12, P_SMR_ = 1.19e‐07, P_HEIDI_ = 1.64e‐01) (Tables S10 and S11). Given that non‐significant P_HEIDI_ values (i.e. *P* ≥ 0.05) indicate an association only because of pleiotropy, both candidates evidenced to affect GI due to pleiotropy rather than linkage.

**Figure 4:**
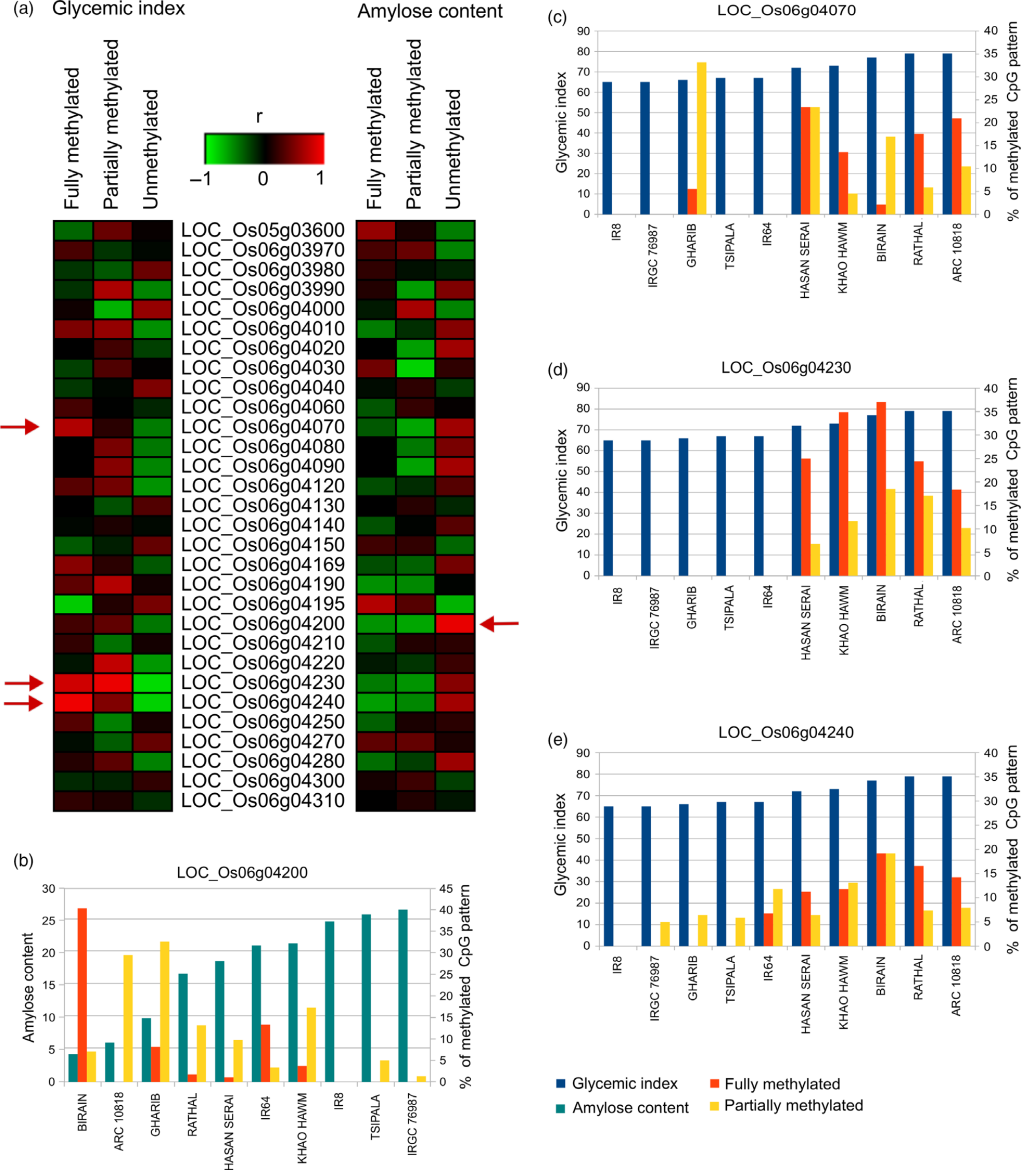
Presence of CpG methylation and their correlation with the GI and amylose content (AC) observed in ten resequenced germplasm lines. (a) Representation of the correlations between degree of CpG methylation present in the promoter region of genes underlying GI6.1 hotspot region with the GI and AC; evaluated in three main categories, fully‐unmethylated, partially (10%–90% methylation) and fully methylated (>90% methylated region) categories. Correlation coefficients ranged from −1 (green) to +1 (red). Horizontal red arrow depicts the candidates showing the significant correlations with the respective trait. (b) Graphs showing the degree of the CpG methylation (fully and partially methylation) patterns in the genic region of the key candidates LOC_Os06g04200 linked with AC, (c) LOC_Os06g04070, (d) LOC_Os06g04230 and (e) LOC_Os06g04240, linked with and GI values (c–e).

Since DNA methylation affects gene expression, we determined the level of methylation of significant genes on GI6.1 through whole genome bisulfite sequencing (WGBS) using ten diverse landraces that belong to different GI/AC classes (Figures 4a–e and S13). We correlated the fully methylated, partially methylated and unmethylated status of genes located in fine mapped GI6.1 genetic region with GI phenotypic values of 10 contrasting lines. We observed high variability in all three methylation contexts (CpG, CHG and CHH) (Figures 4a and S13a). The higher degree of CpG methylation status identified in locus LOC_Os06g4070, LOC_Os06g4230 and LOC_Os06g4240, which is tightly correlated to increase in GI (Figures 4a,c–e and S5). In the case of *GBSSI*, fully methylation did not signify correlation with GI (*r* = 0.26), although higher CpG methylation in *GBSSI* strongly associated with low AC content (*r* = −0.58) (Figures 4a and S13). Two neighbouring CpG islands at the promoter region of *GBSSI* were detected, where the determinant methylation was observed at the upstream CpG islands. Among the three methylation contexts, the partial methylation of CHG had a stronger negative correlation (*r* = −0.94; in *GBSS1* locus) to amylose content compared to either CHH or CpG, while had stronger positive correlation with GI (*r* = 0.89; in locus LOC_Os06g4230) (Figure S13).

### Identifying central hubs from novel metabolic pathways influencing low glycaemic index by gene regulatory network analyses

Gene network analysis conducted using gene expression data were obtained from the 60K microarray resource newly generated from developing grains’ tissues collected during 16 days after fertilization (DAF). These transcriptome data were used to identify GI‐related *trans*‐acting elements in a gene regulatory network formed from a sub‐panel of those with low to intermediate GI and those with intermediate to high GI. We found 156 differentially expressed genes (DEGs) only in the intermediate to high GI lines (haplotypes 1–3, Figure S14a), and also found strong connections between *GBSSI* and the central hub genes where a few were still unannotated (Figure S14b,c). For further details refer Appendix S1.

**Figure 5:**
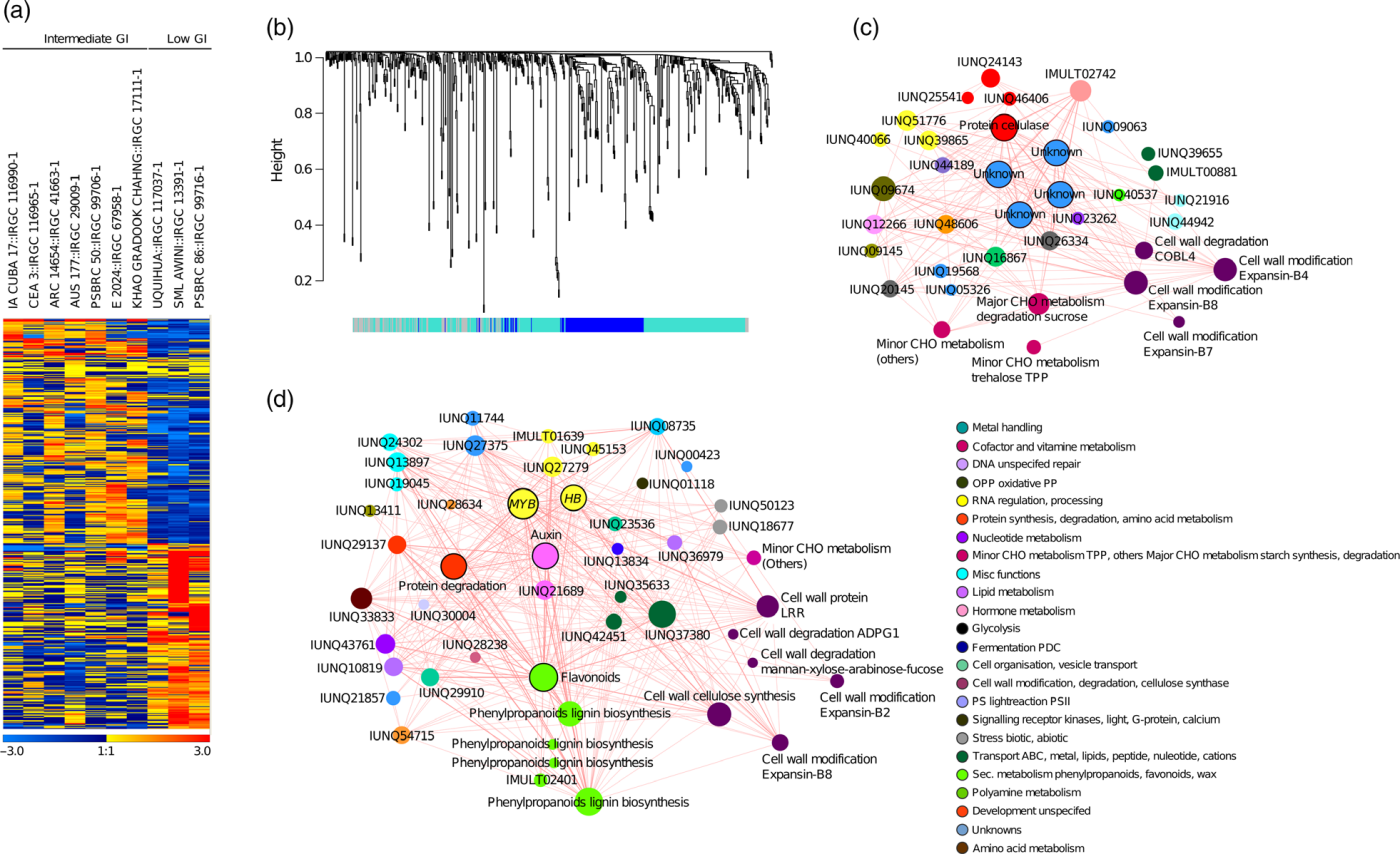
Weighted gene co‐expression analysis among Intermediate and low GI lines. (a) The heat map of differentially expressed genes between the intermediate versus low GI lines (Heatmap scale ranges from min −3 to max +3 normalized expression values which are shown as blue to red colour gradient representing the low and high expression, respectively). (b) Gene dendrogram with two clustered modules blue, turquoise in intermediate versus low; (c, d) Gene co‐expression subnetwork of Blue and Turquoise module, respectively in intermediate versus low. Nodes in each network represent the gene and edges as the interaction, the variation of node size show the different degree of connectivity and bordered wall nodes represents the hub. Nodes are colour coded based on MapMan functional categories.

**Figure 6:**
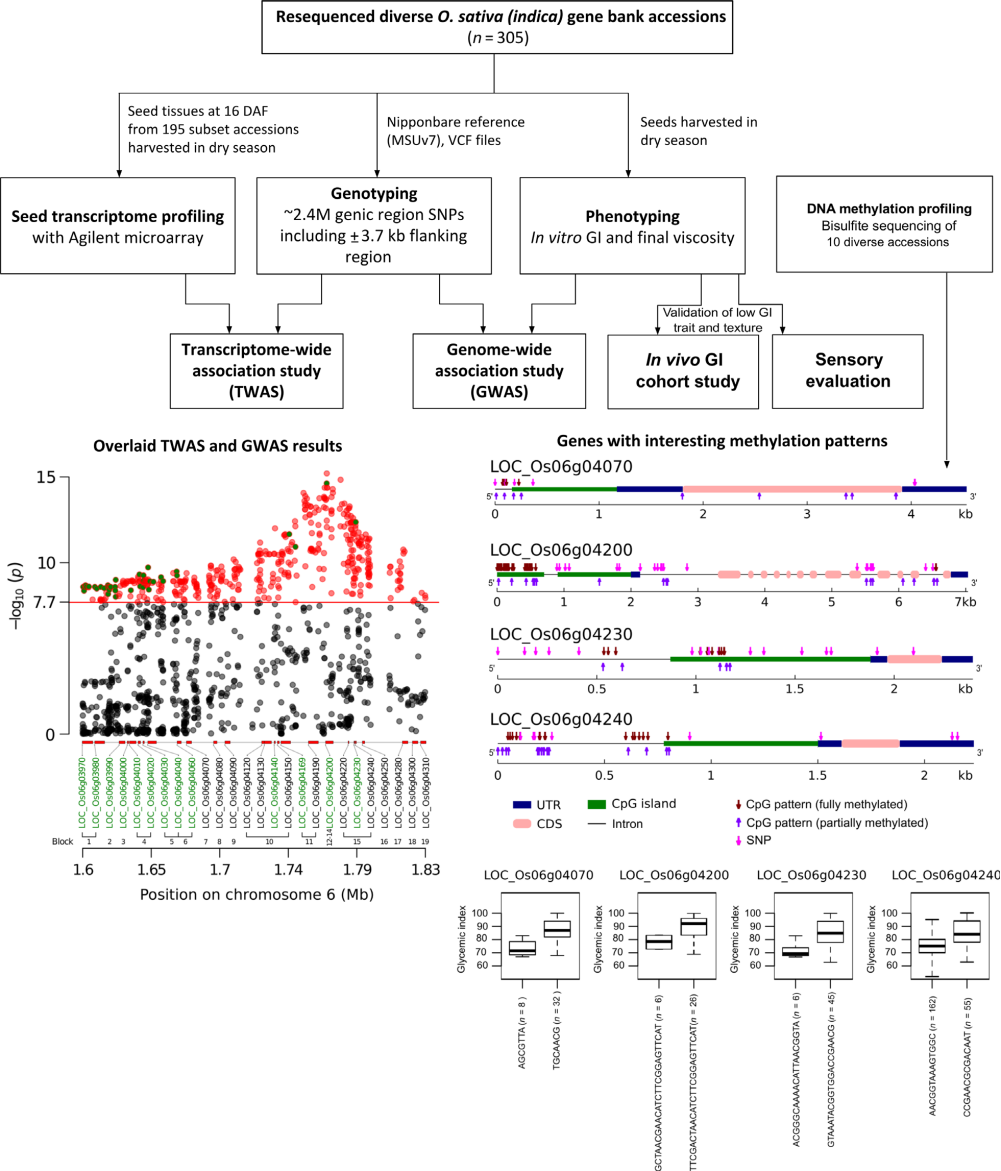
Schematic overview summarizing the predicted regions influencing rice glycaemic index and texture. Overlaying genome‐wide association study with transcriptome‐wide association study results (shown at the bottom left) narrowed the candidate genes down to 13 in the GI6.1 region, and further validated using expression QTL (eQTL) analysis. Four important loci influencing GI trait within GI6.1 region show interesting methylation patterns. The diagnostic haplotypes identified in LOC_Os06g04070, LOC_Os06g04200, LOC_Os06g04230 and LOC_Os06g04240 are critical to lowering the glycaemic index.

The GWAS, TWAS and methylation analysis provided *in silico* evidence about the genetic influence of *GBSS1* and additional unknown genes on the GI phenotype (Figure 6), which were confirmed through gene network analyses. However, this analytical procedure was insufficient to identify the genetic basis of low GI mainly due to the under‐representation of this range of GI values in the panel (Tables S12 and S13). We therefore performed co‐expression network analysis in a panel composed of three low and seven intermediate GI lines. This analysis resulted to the identification of 596 DEGs (Figure 5a) that clustered into two distinct modules (see blue with 142 genes and turquoise with 303 genes in Figure 5b). Among those found at the central hub were a gene that codes for cellulose, while others still have unknown functions (Figure 5c). Substantial differences were observed among the low to intermediate GI lines with respect to cell wall synthesis, lipid metabolism and secondary metabolism pathways (Figure S15a,b). Within the cell wall metabolism pathways, genes related to the biosynthesis of cellulose, cell wall proteins and lignin were up‐regulated (Figure S15a–d), whereas genes involved in the cell wall degradation pathways, mainly non‐starch polysaccharides (NSPs), mannan‐xylose‐arabinose‐fucose and other genes, were down‐regulated (Figures 5d and S15a,b,g and h).

## Discussion

Soft textured low GI rice varieties are important target traits for breeding because many consumers will not consume retrograded hard‐textured rice. The ultimate goal of our research is to define the genetics and identify molecular markers that can be used by marker‐assisted breeding to pyramid ideal alleles into rice varieties with both low GI and acceptable texture. Until now, lowering GI is traditionally linked with developing high amylose mutants and/or transgenics in rice (Asai *et al*., 2014; Butardo *et al*., 2011; Man *et al*., 2013; Zhou *et al*., 2016; Zhu *et al*., 2012), barley (Morell *et al*., 2003) and wheat (Regina *et al*., 2006) by inactivation of *starch branching enzymes* and up‐regulating *GBSSI* genes (Wang *et al*., 2017). As a consequence, increasing amylose content and resistant starch to produce low digestibility rice grain can potentially hamper consumer adoption due to inferior textural attributes of the cooked grain (Butardo and Sreenivasulu, 2016). Since, rice is commonly consumed as intact grains, texture cannot be compromised. In this work, we attempted to identify unique accessions with low *in vitro* GI and softer texture. Instead of using debranched starch structure (Butardo *et al*., 2017), we phenotyped 305 diversity lines for *in vitro* GI for assessing digestibility and starch paste viscosity data as proxy measure for texture. We identified a low GI accession with softer cooked grain texture (GQ02497) by tapping into subtle allelic mutations and recombination's that occurred during the course of rice domestication. We confirmed the low digestibility and soft texture phenotypes through clinical GI and sensory profiling using human volunteers.

### Genetic regions influencing glycaemic index and final viscosity in rice

The *in vitro* digestibility and textural profiles of rice grains were associated with high density SNP coverage (2.4M SNPs) to obtain better mapping resolution using GWAS to identify major effect QTLs associated with these target traits. In this study, we focused on the *indica* subpopulation to remove any confounding influence of population substructure and used rice diversity panel of 305 accessions with amylose content ranging from 0% to 30%. The results further revealed that AC alone is not a good predictor of rice grain digestibility (*r* ^2^ = 0.28) potentially due to contribution of other macromolecular factors influencing cooked rice grain digestion (Butardo and Sreenivasulu, 2016). The correlation coefficient obtained in this study was significantly lower (*r* ^2^ = 0.28) than the previously reported value (*r* ^2^ = 0.73) (Fitzgerald *et al*., 2011), which can be attributed to utility of different sets of IRRI gene bank varieties. The predictive power of AC alone on the GI is very low particularly at the intermediate‐to‐high AC level and thus defining the genetic basis of GI is a prerequisite to initiate future breeding efforts. The first genetic locus detected involves a newly‐identified synonymous T→C SNP in the exon 2 of LOC_Os05g03600 in chromosome 5 with large additive allelic effect correlated with the phenotype conversion from intermediate to high GI.

The second fine mapped 230 kb genetic region termed as GI6.1 located in chromosome 6 harbours 26 genes with a total of 19 linkage blocks, responsible for 88.7% of the total variation in GI. The rate of the LD decay was found to be faster in GI6.1, indicating very high recombination rate in this hotspot region. This result is corroborated by Olsen *et al*. (2006) who previously identified a ~250 kb block that experienced a high selection pressure during the long history of rice domestication. *GBSSI* gene has an unusually high recombination rate, with various alleles preferentially selected during rice domestication which represent the diverse cooked grain texture and reflect the diverse cultural preference of rice consumers in Asia. Interestingly, two linkage blocks were detected within the *Waxy* locus with an unusual LD decay. The first SNP (T→G) at the 5′end of intron 1 is known to reduce the expression of *GBSS1* in developing endosperm (Cai *et al*., 1998; Isshiki *et al*., 1998; Larkin and Park, 1999; Okagaki, 1992; Wang *et al*., 1995). This leads to glutinous to low amylose phenotype. Rice grains of this type are known to have high digestibility due to the presence of elevated proportion of highly branched amylopectin in their starch fraction. The second point mutation (C→T SNP) in exon 10 does not influence the GI but is significantly associated with FV, affects the rice cooking and eating quality of rice (Cuevas and Fitzgerald, 2012; Kharabian‐Masouleh *et al*., 2012; Larkin and Park, 2003). This C→T SNP was also detected prominently when GWAS was conducted on FV using AC as covariate, further validating the results. In addition, a significant SNP was located in the linkage break between the two LD blocks within the *Waxy* locus. The SNP is an A→C SNP (snp_06_1768006) that influences the GI, located two base pairs towards the 3′ end of exon 6 (Figure S4b). This SNP has been previously reported to discriminate between intermediate and high AC varieties (Chen *et al*., 2008; Larkin and Park, 2003; Mikami *et al*., 2008). All these results highlight the importance of *GBSSI* in influencing digestibility to convert high GI into intermediate GI with soft and hard textured rice.

### Inferring the relevance of key GBSS SNPs during the course of rice domestication that influence the digestibility and textural traits by phylogenetic analysis

Haplotype analyses based on the two highly discriminating *GBSSI* SNPs; the splice junction SNP T→G was found relevant for converting high to intermediate GI and the exon 10 SNP C→T for converting intermediate to high FV. The 2 SNP based haplotype combination influencing GI and FV was able to classify rice accessions into culturally similar regions based on phylogenetic analysis. South Asian countries prefer hard/fluffy textured rice which possess GC or GT haplotypes, where the G allele (*Wx* ^*a*^) and the T allele explains preference for high amylose and harder texture in the region, respectively (Chen *et al*., 2008; Larkin and Park, 2003). In contrast, Southeast Asian countries have stronger preference for softer rice, which possess TC haplotype with characteristic feature of intermediate amylose and low‐to‐intermediate FV. Rice accessions in these countries have low‐to‐intermediate FV and high GI mainly due to the activity of the *Wx* ^*b*^ allele leading low to intermediate AC. These results add to the growing body of evidence that supports the complex domestication of rice that is influenced by regional cultural preferences (Calingacion *et al*., 2014; Civáň *et al*., 2015; Sweeney and McCouch, 2007). Furthermore, the low GI line GQ02497 with haplotype GC defined within *GBSS1* (with low FV value) is unique for textural attributes such as stickiness between grains and higher starch coating and is distinguishable from other two hard textured low GI lines GQ01795 and GQ02522 with haplotype GT (Tables S7 and S8). The rare rice germplasm GQ02497 with low GI and soft texture can be used as pre‐breeding material to develop low GI rice. It will otherwise remain undetected unless integrative systems genetics approaches is employed, as will be elaborated below.

### Gene regulatory mechanisms linked to phenotype of lowering glycaemic index in GI6.1 region

To decipher the gene regulatory mechanisms of candidate genes involved in the GI response, we conducted TWAS analyses using *GBSSI* probe of transcriptomic data generated from 195 *indica* rice lines. This resulted in the identification of important SNP involved in alternative splicing of *GBSSI*, as well 13 other genes belongs to different linkage blocks within GI6.1 predicted to influence GI phenotype through *cis* effect. Furthermore, SMR analysis was conducted to differentiate between pleiotropic and linkage effects, and to provide corroborative statistical evidence of the functional relevance of genes including *GBSSI* and LOC_Os06g04169 (encoding transmembrane glycosyl hydrolase) acted through *cis* effect within GI6.1 genetic region. Two hydrolase genes from separate linkage blocks of GI6.1 were detected to have non‐synonymous SNP mutations which can potentially influence digestibility through glycosyl hydrolase and LOC_Os06g04080 encoding β‐1,3 glucan hydrolases. Glycosyl and glucan hydrolases digest β‐glucans, which are the main component of cell wall‐derived NSPs in cereal grains (Fincher, 2009; Houston *et al*., 2014). Changes in the protein structure through identified SNP based non‐synonymous mutations potentially lower the digestive efficiency of these hydrolases in metabolizing β‐glucans in the rice cell wall, which alters the GI. In addition, the gene regulatory networks derived based on co‐expression analyses of low GI versus intermediate GI lines explained the slow digestibility associated with the cell wall metabolism pathways, where genes related to the biosynthesis of NSPs were significantly up‐regulated while the genes involved in NSP degradation were down‐regulated in low GI lines. It appears that the low GI rice varieties favoured the carbon sink for NSPs related to the cell wall residues in endosperm that can potentially reduce digestibility (Lafiandra *et al*., 2014; Topping and Clifton, 2001). An intact cell wall with higher dietary fibre upon cooking is known to lower the *in vitro* digestion of legumes (Dhital *et al*., 2016), and a similar mechanism might exist in rice (Butardo *et al*., 2017; de Guzman *et al*., 2017). NSP is well‐known contributor to dietary fibre (Tungland and Meyer, 2002) and is important in lowering the GI of food. Our multi‐regulatory ‐omics analyses further strengthen the concept that apart from amylose, NSPs can also affect digestibility.

In addition, genes encoding the histone H1 (LOC_Os06g04020), H3 (LOC_Os06g04030) and GAGA binding protein genes involved in chromatin restructuring, likely regulating the expression of *GBSS1* were identified through TWAS. GAGA transcription factor was reported to be associated with heterochromatin formation and nucleosome remodelling in target genes (Adkins *et al*., 2006). Both H1 and H3 are required for the packing of chromosomal DNA into highly compact chromatin, which adversely affects the binding of transcription factors. To test the role of DNA methylation in regulating *GBSSI* expression and additional 13 genes identified in GI6.1 genetic region, we have subjected ten diverse landraces shown to possess diverse GI and AC phenotypic values. Within GI6.1 region, low methylation of *GBSSI* promoter region was strongly associated with high amylose content. The presence of two neighbouring CpG islands at the promoter region of *GBSSI*, where the determinant methylation mentioned above was also observed at the upstream CpG island(s). Another interesting result was that while *GBSSI* promoter methylation was moderately correlated to influence GI, we identified lower methylation of promoter region at locus LOC_OS06g04070 and genes sitting downstream of *GBSSI* loci namely LOC_OS06g04230 and LOC_OS06g04240 were strongly correlated to decrease the GI (refer Figure 6). This suggests some of the regulatory elements involved in a methylation‐based transcription regulation of GI, is through posttranscriptional regulation of *GBSSI* as well as, other unknown genes situated in neighbourhood loci influence GI. Further study is warranted to functionally validate the role of these regulatory elements in conferring reduced digestibility.

## Conclusions

A low GI rice variety with hard texture is undesirable in South East Asian market niches, where soft and sticky types are the benchmarks of rice quality. Our analytical pipeline linking GWAS with TWAS/SMR analysis, epigenomics and transcriptome analysis have pointed to complex regulation of *GI6.1* region, shown to influence GI as a regulon via *cis* effects. This fine‐mapped hotspot region in chromosome 6 harbour key genes such as *GBSSI* that potentially play specific roles in the mediating high GI to intermediate GI through alternative spliced SNP sitting in exon 1 and another in exon 10 influencing lower FV (which is independent from GI and AC influence). Thus recombining two SNP based GC haplotype shown to be preferentially selected during domestication to capture consumer preferences of South East Asia is useful for future molecular marker‐assisted breeding to capture textural preferences. This study focused on the strengths of natural variation occurred over several thousand years identified superior germplasm with low GI and soft texture, explaining recombination of several beneficial alleles for lowering GI with soft texture through multi‐OMICS studies. Validating these results through functional characterization would be a daunting task, requires targeting many more genes to recombine several superior alleles. Hence functional validation was not prioritized for this study. Lowering GI is being linked to non‐synonymous mutations in hydrolase gene identified as significant in SMR analysis in influencing GI, likely slowing digestibility through NSPs. Lastly, we also deciphered post‐regulatory mechanism influencing the methylation status of a set of unknown genes LOC_OS06g04070, LOC_OS06g04230 and LOC_OS06g04240 located within *GI6.1* which strongly influences the GI phenotype. Such intensive allele recombination that happened during domestication in fine mapped *GI6.1* is an important source to select superior alleles for combined low GI with soft texture through systems‐genetics concept validated using *in vivo* human clinical studies and descriptive sensory profiling done by human cohorts. Unique accessions identified in this study are currently being used as pre‐breeding materials to develop low GI lines targeted towards certain market segments, particularly in Asia.

## Experimental procedure

### Plant materials and phenotyping

The plant materials used in this study included 305 indica varieties randomly selected from the indica varieties in the 3000 rice genomes that are known to mature within 140 days (refer the Table S12 and Appendix S1). The rice was planted in three replicates using a complete block design at the IRRI experimental field during the 2015 dry season. Milled rice samples were used to screen the digestibility of the rice lines using *in vitro* glycaemic index (GI) method. The GI was measured at the Commonwealth Scientific and Industrial Research Organisation (CSIRO), Adelaide, Australia using a predictive *in vitro* protocol that mimics the oral, gastric, pancreatic and intestinal digestion process in the human gut. The *in vitro* system uses a cocktail of enzymes, including human and porcine alpha‐amylases, pepsin, pancreatin and amyloglucosidase, to very effectively model the process of starch assimilation in the human upper gastrointestinal tract. The method used by the CSIRO laboratory to predict the food GI has been validated against data obtained from clinical studies in humans. This *in vitro* digestibility method has been substantially tested in diverse rice lines (Butardo *et al*., 2011).

The final viscosity (FV) was measured using a rapid viscosity analyzer (RVA) at the IRRI Grain Quality Nutrition and Services Laboratory (IRRI GQNSL) following the described standard method for rice (Butardo *et al*., 2017). The high‐throughput amylose content (AAC) estimation was also performed as previously described (Butardo *et al*., 2017).

*In vivo* GI of two milled rice accessions (GQ02497 and GQ02522) was evaluated using standardized protocol (Trinidad *et al*., 2013) at the Food and Nutrition Research Institute (FNRI), Department of Science and Technology (DOST), Philippines. A cohort study with at least 12 human subjects was undertaken to determine the mean GI values (for details, refer to Appendix S1).

### Genome‐wide association study and gene set analysis

The single nucleotide polymorphism (SNP) genotype data used in this study was obtained from the 305 re‐sequenced genomes mapped against the Nipponbare reference genome (MSU7). The variant call files (VCF) were downloaded from the IRRI local repository and processed using bcftools retaining only the high‐quality biallelic SNPs with minimum quality score of 30 and sequencing depth between 4 and 70. Population structure was calculated using fastStructure, while linkage disequilibrium and haplotype blocks were calculated using plink (refer to Appendix S1).

A total of 2,419,731 high‐quality SNPs from the 305 *indica* panel with 99.2% genotyping rate and minor allele frequency cut‐off of 5% were associated with both the GI and FV phenotyping data in a mixed linear model GWAS using efficient mixed‐model association expedited (EMMAX) (Kang *et al*., 2010). Prior to performing the GWAS, both phenotypes were transformed using the warped linear mixed model (WarpedLMM) software (Fusi *et al*., 2014). The Balding‐Nichols kinship matrix (Balding and Nichols, 1995) was calculated using the EMMAX‐KIN.

Once the association results were complete, we calculated both the Bonferroni‐corrected *P*‐values using the stats::p.adjust() function in *R* and the *q*‐values using the qvalue::qvalue() function in R/Bioconductor. The GWAS significance threshold was set at the Bonferroni‐corrected α′ = 2.066345e‐08 calculated as α′ = 0.05/m, where m was the total number of SNP markers. The results were shown in –log scale in both the Manhattan and Q‐Q plots. We highlighted SNPs with *q*‐value<0.05 in the Manhattan plot to indicate other regions that could potentially be associated with the trait had our experimental design have sufficient statistical power to detect minor QTLs.

We performed a gene set analysis using the publicly available MAGMA software (de Leeuw *et al*., 2015) on those genes implicated by Bonferroni‐significant SNPs to further narrow down the candidates. Internal correction for multiple testing was inherent in the software thus we used a significance threshold of *P* < 0.05.

### Transcription‐wide association study and summary‐based Mendelian randomization analysis

We performed a summary‐based Mendelian randomization (SMR) analysis (Zhu *et al*., 2016) to establish causality due to pleiotropic effect of the *cis*‐elements on both the gene expression and the phenotype. This analysis utilized the result of a transcription‐wide association study (TWAS), where we only focused on those genes that came out significant after GWAS and gene set analysis. To be consistent, the *cis*‐region was defined as a narrow 1 kb region both upstream and downstream of the gene. This definition was used across GWAS, TWAS, *cis*‐eQTL and SMR analyses.

Expression data were obtained from developing grains collected at 16 days after fertilization (DAF) from 195 lines in the same indica panel used for GWAS. The grain tissues collected were rapidly frozen in liquid nitrogen, homogenized, subjected to RNA isolation using Qiagen RNeasy Plant Mini Kit, testing the RNA integrity number with 7.0 or above and subjecting it to cDNA synthesis and cRNA labelling using a single‐colour Low Input Quick Amp Labelling Kit, hybridizing the labelled probe to 60K indica microarrays in SureHyb chamber and scanning the microarrays (with an ozone barrier slide) using SureScan Microarray Scanner following the methods described in Butardo *et al*. (2017). The expression profiles were quantile‐transformed in using limma (Ritchie *et al*., 2015) a package in R/Bioconductor package prior to usage in the TWAS pipeline. The TWAS had a slightly reduced number of SNPs, i.e., 2 375 601, mainly due to the reduced number of samples. However, the resolution was sufficiently high for the genome‐wide interrogation. The significance threshold was also a Bonferroni‐corrected α < 0.05, which was calculated in the same way as that for the GWAS. Detailed protocols of microarray based transcriptome analysis and methods employed to study gene regulatory networks highlighted in Appendix S1.

### Methylation analysis

For further details refer the Appendix S1.

### Texture and sensory evaluation

For further details refer the Appendix S1.

## Conflict of interest

The authors declare no conflict of interest.

## Supporting information


**Figure S1** Schematic representation of the study conducted.
**Figure S2** Linkage disequilibrium (LD) decay.
**Figure S3** Distribution of haplotype groups originating from different LD‐blocks within the GI6.1 region.
**Figure S4** Linkage disequilibrium involving the *granule‐based starch synthase I* (*GBSS I*) and neighbouring genes.
**Figure S5** Gene structure model with distribution of SNP and methylation pattern in the candidates identified through targeted gene association study.
**Figure S6** Phenotypic variation explained by haplotypes formed by significant SNPs in *granule bound starch synthase I* (*GBSS I*).
**Figure S7** Circular unrooted neighbour‐joining phylogenetic tree calculated using MEGA7.
**Figure S8** Plots of the rapid viscosity analyzer (RVA) profiles of the diversity panel.
**Figure S9** Summary for SNPs that are within the promoter and (−1 kb) promoter region of a gene encoding transmembrane glycosyl hydrolase (LOC_Os06g04169).
**Figure S10** Phenotypic variation explained by haplotypes formed by significant SNPs in a gene encoding transmembrane glycosyl hydrolase (LOC_Os06g04169).
**Figure S11** Genome‐wide association study for glycemic index with amylose content as covariate.
**Figure S12** Genome‐wide association study for final viscosity (FV) with amylose content (AC) as covariate.
**Figure S13** Correlation of GI and AC with the level of methylation existing in the genic region of the genes underlying *GI6.1* hotspot region, in the ten resequenced lines.
**Figure S14** Weighted gene co‐expression analysis among high and intermediate GI lines.
**Figure S15** Overview of gene expression profile by MapMan.

**Table S1** Evaluation of two accessions (GQ02522 and GQ02497) for *in vivo* GI values using human clinical study.
**Table S2** Comparison of textural attributes of Dinorado evaluated in two panel sessions using the *t*‐test (*t*) or the Wilcoxon Rank Sum (W) test.
**Table S3** Comparison of textural attributes of low‐GI samples GQ02522 and Dinorado as evaluated by sensory panelists, using the *t*‐test.
**Table S4** Comparison of textural attributes of low‐GI samples GQ01795 and Dinorado as evaluated by sensory panelists, using the *t*‐test.
**Table S5** Comparison of textural attributes of low‐GI samples GQ02497 and Dinorado as evaluated by sensory panelists, using the *t*‐test.
**Table S6** Comparison of hardness, springiness, adhesiveness and cohesiveness, obtained by texture profile analysis (TPA).
**Table S7** Genome‐wide association study (GWAS) on glycemic index.
**Table S8** Genome‐wide association study on glycemic index with amylose content as covariate.
**Table S9** Genome‐wide association study on final viscosity with amylose content as covariate.
**Table S10** eQTL analysis on the Waxy locus.
**Table S11** Functionally relevant genes as evidenced by genome‐wide association study (GWAS), cis‐eQTL, summary‐based Mendelian randomization (SMR), and heterogeneity in dependent instruments (HEIDI) analyses.
**Table S12** List of 305 accessions used in the GWAS study.
**Table S13** Detail of accessions used in the gene regulatory network analysis.

**Appendix S1** Supporting experimental procedures.
