# Supplementary material for: Integrating a genome‐wide association study with a large‐scale transcriptome analysis to predict genetic regions influencing the glycaemic index and texture in rice
Source: Plant Biotechnol J. 2019 Jan 24;17(7):1261–75. doi: 10.1111/pbi.13051 (PMC6575982; doi:10.1111/pbi.13051)
Supplement: Supplementary file 1 — Figure S1 Schematic representation of the study conducted. Figure S2 Linkage disequilibrium (LD) decay. Figure S3 Distribution of haplotype groups originating from different LD‐blocks within the GI6.1 region. Figure S4 Linkage disequilibrium involving the granule‐based starch synthase I (GBSS I) and neighbouring genes. Figure S5 Gene structure model with distribution of SNP and methylation pattern in the candidates identified through targeted gene association study. Figure S6 Phenotypic variation explained by haplotypes formed by significant SNPs in granule bound starch synthase I (GBSS I). Figure S7 Circular unrooted neighbour‐joining phylogenetic tree calculated using MEGA7. Figure S8 Plots of the rapid viscosity analyzer (RVA) profiles of the diversity panel. Figure S9 Summary for SNPs that are within the promoter and (−1 kb) promoter region of a gene encoding transmembrane glycosyl hydrolase (LOC_Os06g04169). Figure S10 Phenotypic variation explained by haplotypes formed by significant SNPs in a gene encoding transmembrane glycosyl hydrolase (LOC_Os06g04169). Figure S11 Genome‐wide association study for glycemic index with amylose content as covariate. Figure S12 Genome‐wide association study for final viscosity (FV) with amylose content (AC) as covariate. Figure S13 Correlation of GI and AC with the level of methylation existing in the genic region of the genes underlying GI6.1 hotspot region, in the ten resequenced lines. Figure S14 Weighted gene co‐expression analysis among high and intermediate GI lines. Figure S15 Overview of gene expression profile by MapMan. [file PBI-17-1261-s002.pdf]

Figure S1

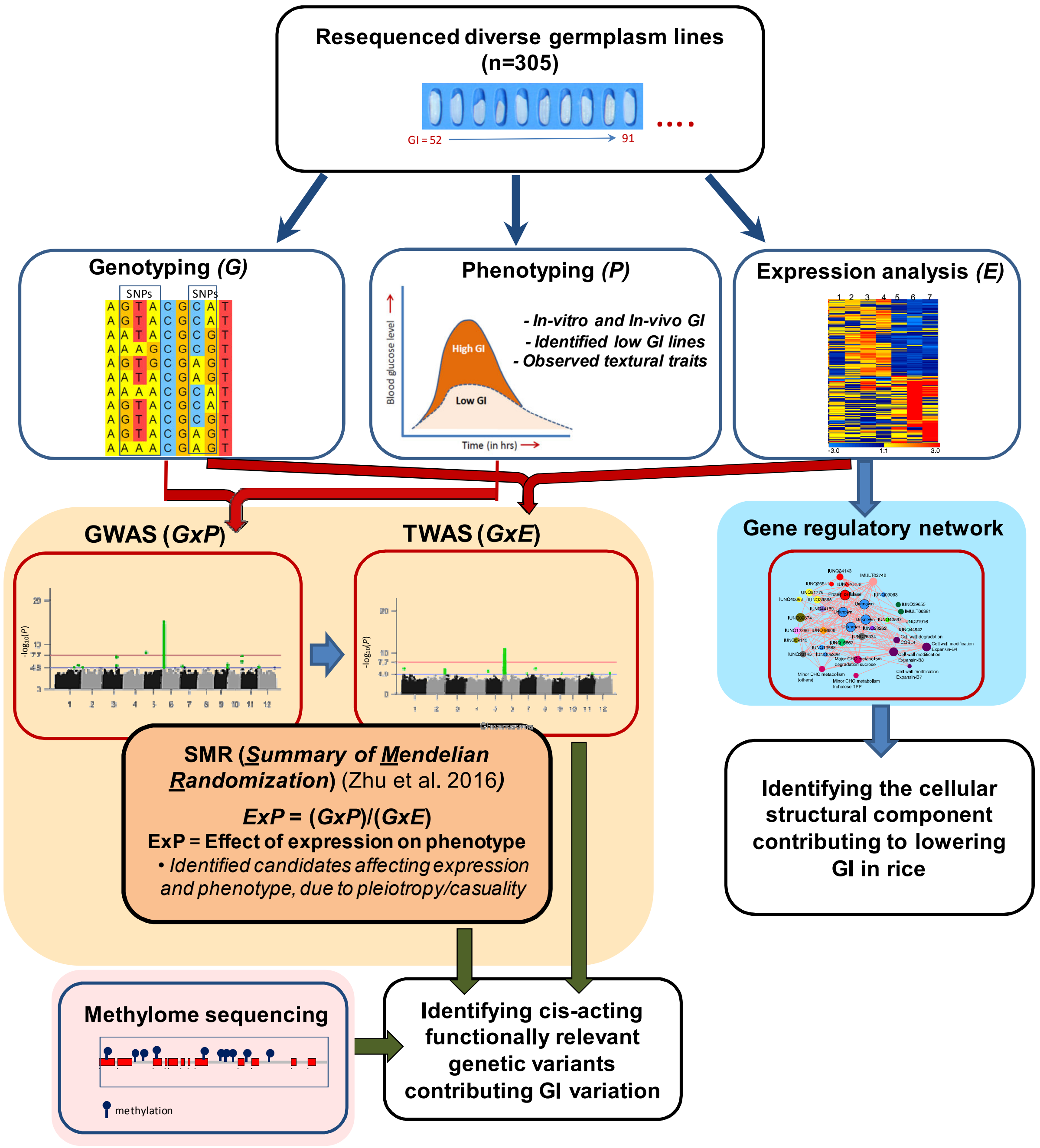

Figure S1: Schematic representation of the study conducted.

Figure S2

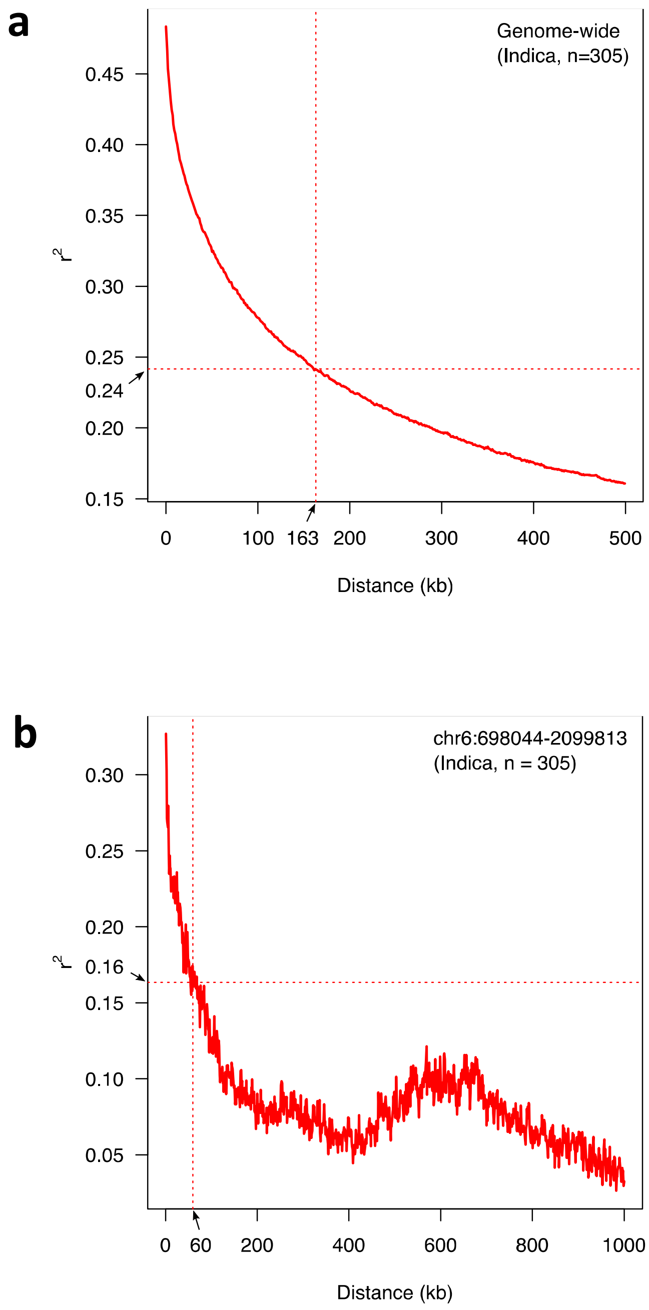

Figure S2 Linkage disequilibrium (LD) decay. (**a**) Genome-wide average LD decay calculated using 307,903 approximately equally spaced markers systematically selected one marker per 500 base pairs for 305 individuals. The average r^2 dropped to 0.24 that was half its maximum value at chromosomal distance 163 kb. (**b**) Long-range average LD decay calculated for the region where the association signal was found. The average r^2 dropped to 0.16, which was half its maximum value for this specific region, at a chromosomal distance of 60 kb.

Figure S3

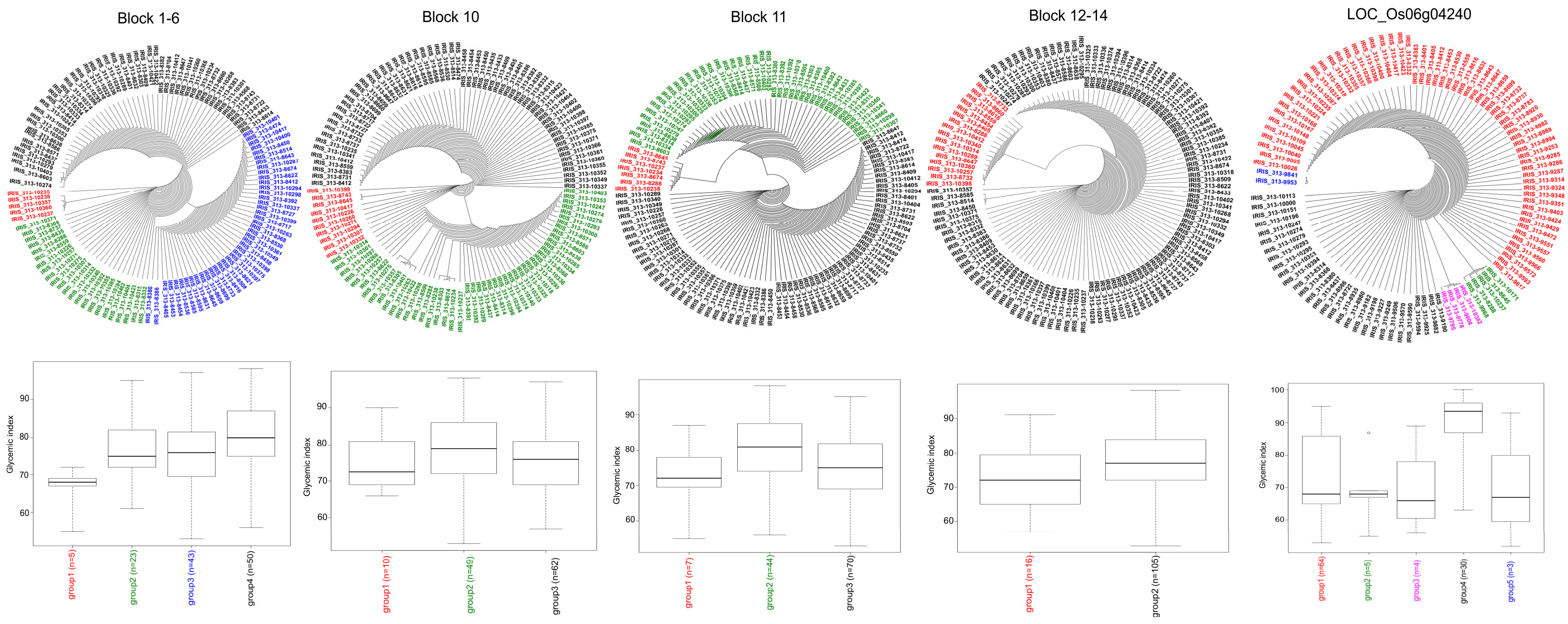

Figure S3: Distribution of haplotype groups originating from different LD-blocks with in the GI6.1 region. The dendrograms and boxplots of different groups were constructed based on the significant SNPs from LD-block 1 to 6 (a), block 10 (b), block 11(c), block 12 to 14 (d) and candidate LOC_Os06g04240 (e). Haplotypes in the boxplots were represented on the basis of influence of respective haplotype on the GI values. Number of the accessions bearing the haplotype groups are mentioned in parentheses.

Figure S4

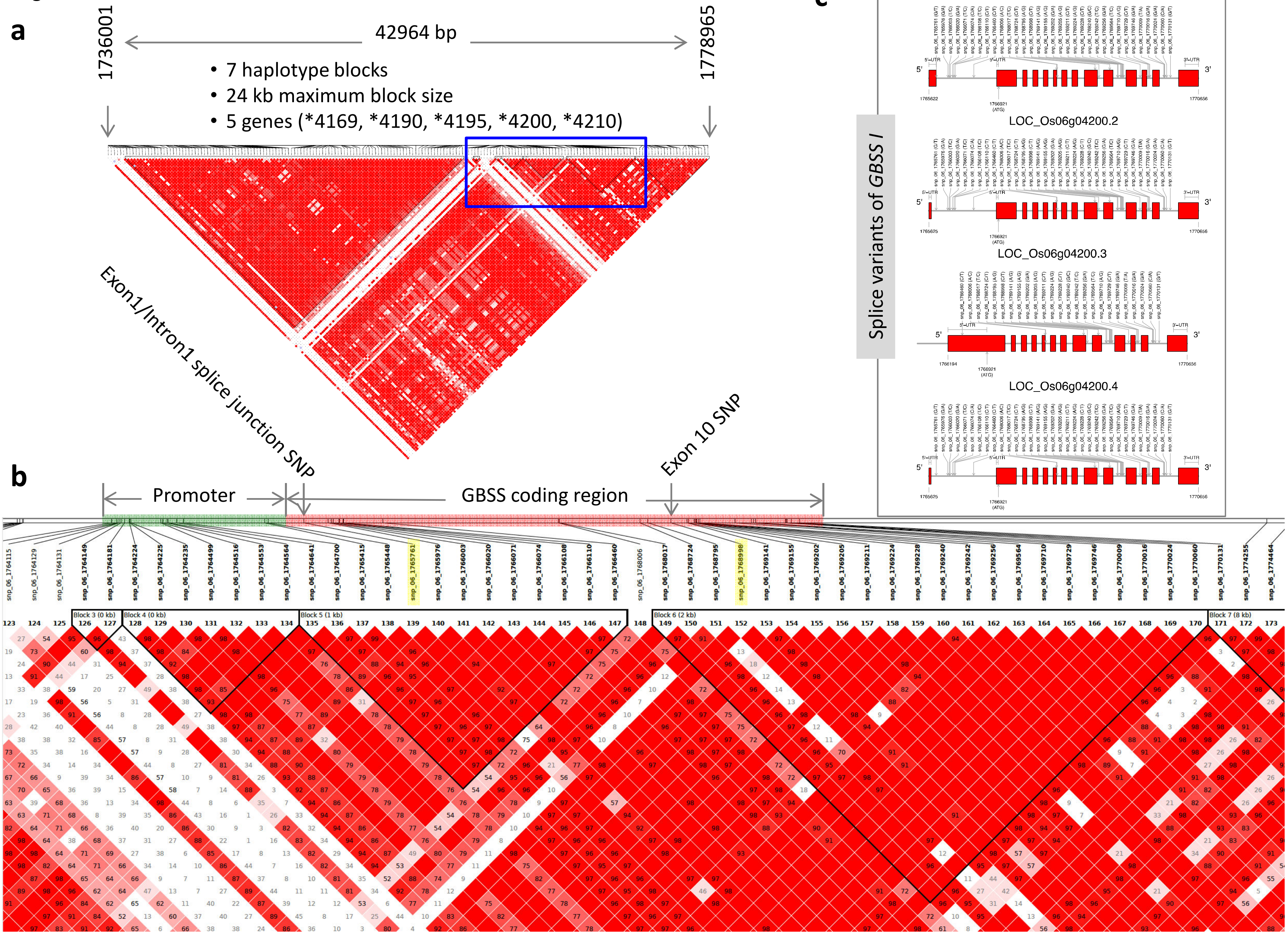

Chromosome 6
a
1736001
42964 bp
1778965
• 7 haplotype blocks
• 24 kb maximum block size
• 5 genes (*4169, *4190, *4195, *4200, *4210)
Exon1/Intron1 splice junction SNP
Exon 10 SNP
b
Promoter
GBSS coding region
c
Splice variants of GBSS I
LOC_Os06g04200.1
LOC_Os06g04200.2
LOC_Os06g04200.3
LOC_Os06g04200.4

Figure S4. Linkage disequilibrium involving the *granule-based starch synthase I* (*GBSS I*) and neighboring genes. (**a**) Calculation of haplotype blocks using Haploview 4.2 within the region Chr6:1736001-1778965 calculated using Gabriel's algorithm implemented in the software with 95% confidence interval on D', and minimum minor allele frequency of 0.05. Only significant SNPs were considered and the five genes where these SNPs were found. The limits were chosen purposely to include the LOC_Os06g04169 (encoding transmembrane glycosyl hydrolase), *GBSS I*, and the gene immediately downstream to visualize the linkage disequilibrium between these genes. The * is an abbreviation of "LOC_Os06g0". (**b**) The detailed view of the region marked by a blue box in (a) that corresponds to *GBSS I*. The chromosome segment marked by green represents the promoter region of *GBSS I* 1 kb upstream of the transcription start site. The chromosome segment marked by red represents the coding region of *GBSS I*. The locations where the splice junction SNP and the exon 10 SNP were located were marked by arrows in their corresponding haplotype blocks. (**c**) The gene structure of the four splice variants of the *GBSS I* showing the complete set of available genic SNPs.

Figure S5

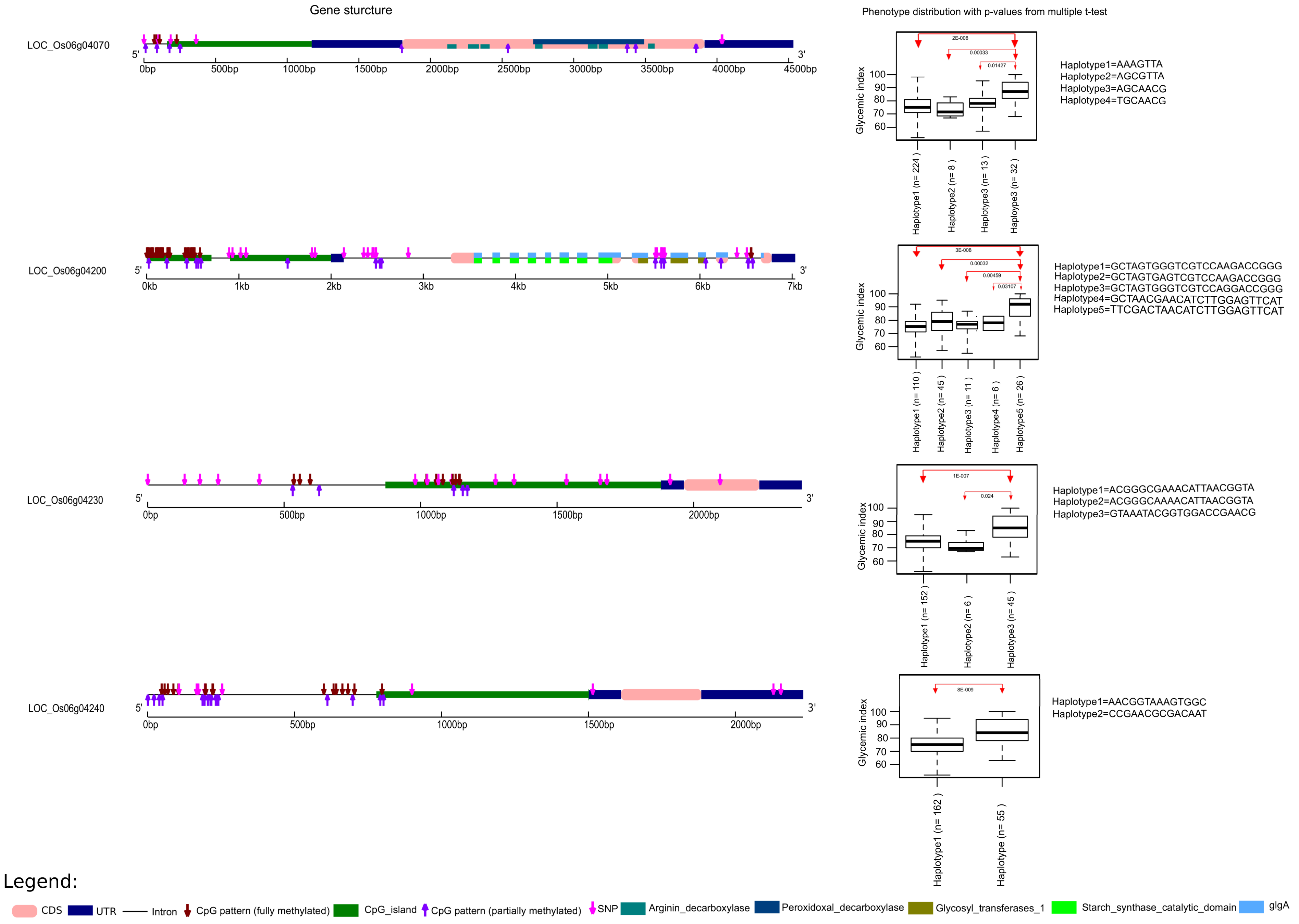

Gene sturcture
Phenotype distribution with p-values from multiple t-test
LOC_Os06g04070
0bp 500bp 1000bp 1500bp 2000bp 2500bp 3000bp 3500bp 4000bp 4500bp
2E-008
0.00033
0.01427
Glycemic index
Haplotype1 (n= 224)
Haplotype2 (n= 8)
Haplotype3 (n= 13)
Haplotype3 (n= 32)
Haplotype1=AAAGTTA
Haplotype2=AGCGTTA
Haplotype3=AGCAACG
Haplotype4=TGCAACG
LOC_Os06g04200
0kb 1kb 2kb 3kb 4kb 5kb 6kb 7kb
3E-008
0.00032
0.00459
0.03107
Haplotype1 (n= 110)
Haplotype2 (n= 45)
Haplotype3 (n= 11)
Haplotype4 (n= 6)
Haplotype5 (n= 26)
Haplotype1=GCTAGTGGGTCGTCCAAGACCGGG
Haplotype2=GCTAGTGAGTCGTCCAAGACCGGG
Haplotype3=GCTAGTGGGTCGTCCAGGACCGGG
Haplotype4=GCTAACGAACATCTTGGAGTTCAT
Haplotype5=TTCGACTAACATCTTGGAGTTCAT
LOC_Os06g04230
0bp 500bp 1000bp 1500bp 2000bp
1E-007
0.024
Haplotype1 (n= 152)
Haplotype2 (n= 6)
Haplotype3 (n= 45)
Haplotype1=ACGGGCGAAACATTAACGGTA
Haplotype2=ACGGGCAAAACATTAACGGTA
Haplotype3=GTAAATACGGTGGACCGAACG
LOC_Os06g04240
0bp 500bp 1000bp 1500bp 2000bp
8E-009
Haplotype1 (n= 162)
Haplotype (n= 55)
Haplotype1=AACGGTAAAGTGGC
Haplotype2=CCGAACGCGACAAT
Legend:
CDS
UTR
Intron
CpG pattern (fully methylated)
CpG_island
CpG pattern (partially methylated)
SNP
Arginin_decarboxylase
Peroxidoxal_decarboxylase
Glycosyl_transferases_1
Starch_synthase_catalytic_domain
glgA

Figure S5: Gene structure model with distribution of SNP and methylation pattern in the candidates identified through targeted gene association study. The distribution of CpG pattern including fully methylation (downward brown arrow) and partially methylation (upward blue arrow) in the genic region of four candidates LOC_Os06g04070, LOC_Os06g04200, LOC_Os06g04230 and LOC_Os06g04240 along with SNP distribution (downward pink arrow) identified from GWAS contributing to distinguish GI phenotypes are presented. Rest of the color coding showed in the gene structure model is shown as footer of the figure. On the right hand side, haplotypes constructed from the respective set of SNPs were represented in the boxplot with their effect on the GI value (representing lines are mentioned in the parentheses). In boxplots, haplotypes showing their significance over other haplotype(s) (at the significance level of $P \leq 0.05$ using pair wise t-test) were marked in red arrows.

Figure S6

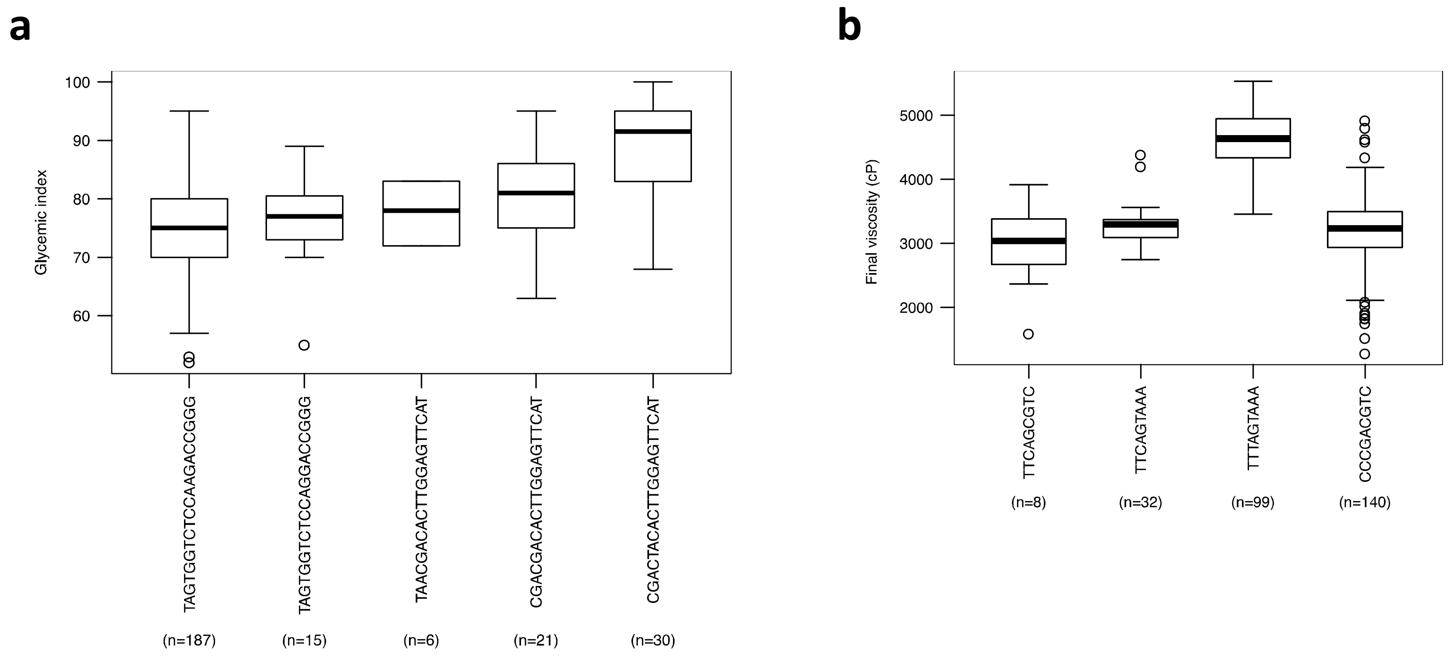

Figure S6 Phenotypic variation explained by haplotypes formed by significant SNPs in *granule bound starch synthase I* (*GBSS I*). (**a**) A total of 20 significant SNPs in the promoter (-1 kb) and genic regions of *GBSS I* (see **Fig. 3a**) explaining phenotypic variation of glycaemic index (GI). (**b**) Variation in final viscosity (FV) explained by nine SNPs in *GBSS I* that have p<1e-20 (see **Fig. 3a**).

Figure S7

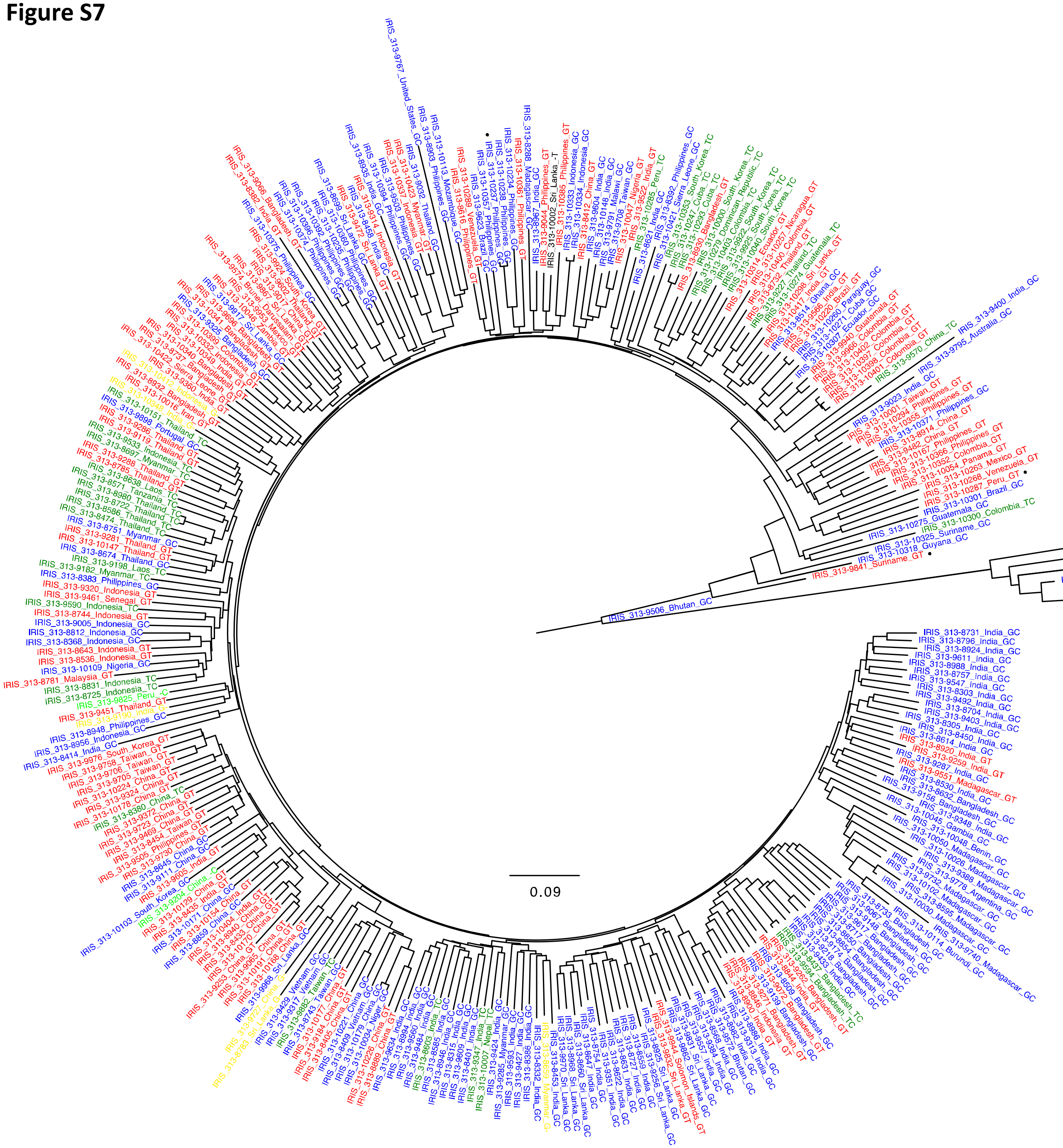

Figure S7 Circular unrooted neighbor-joining phylogenetic tree calculated using MEGA7. In the phylogenetic tree, the colors of the labels represent the haplotype groups. Suffixed in the variety ID is the name of the country source of the germplasm followed by two-SNP haplotype. Scale of the genetic distance is shown in the middle lower region of the inner circle.

Figure S8

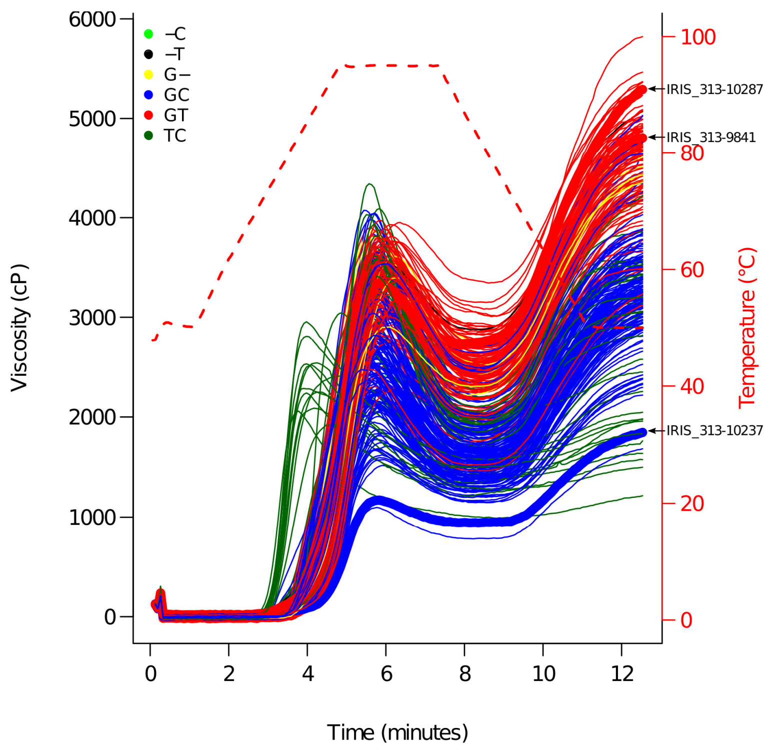

Figure S8 : Plots of the rapid viscosity analyzer (RVA) profiles of the diversity panel. In the RVA-plot, colored curves represent the GT (red), GC (blue) and TC (green) haplotypes (color legend mentioned at upper right corner of the figure). Light green, black and yellow curves represent those with missing genotype calls in either of the two represented SNPs. The red dotted line as per the secondary Y-axis, represents the temperature variation throughout the entire RVA run. Plot for three low GI accessions were highlighted as thicker (with higher weight) curves, while colored based on their presence of the haplotype group.

Figure S9

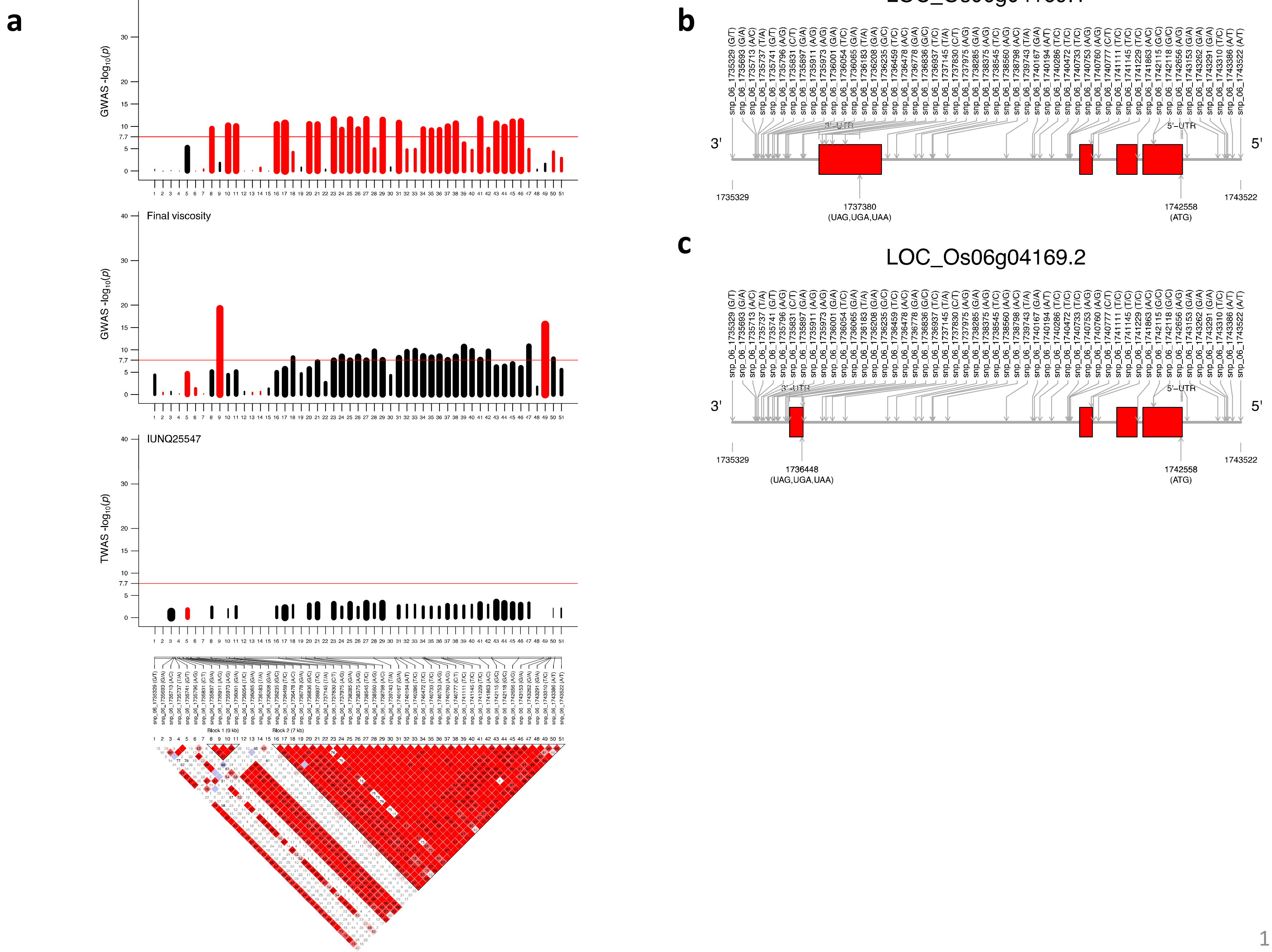

a
Glycemic index
GWAS -log10(p)
Final viscosity
GWAS -log10(p)
IUNQ25547
TWAS -log10(p)
Block 1 (0 kb)
Block 2 (7 kb)
b
LOC_Os06g04169.1
3'
5'
3'-UTR
5'-UTR
1735329
1737380
(UAG,UGA,UAA)
1742558
(ATG)
1743522
c
LOC_Os06g04169.2
3'
5'
3'-UTR
5'-UTR
1735329
1736448
(UAG,UGA,UAA)
1742558
(ATG)
1743522

Figure S9 Summary for SNPs that are within the promoter and (-1 kb) promoter region of a gene encoding transmembrane glycosyl hydrolase (LOC_Os06g04169). (**a**) The topmost track shows the $–\log_{10}$ plot of the association *p*-values for glycemic index (GI). A red bar indicates that the effect allele of the SNP causes a decrease in GI while a black bar is the opposite. Bar thickness is reflective of the relative effect size of the effect allele compared to the rest of the SNPs in the gene. Below the topmost track is the $–\log_{10}$ plot of the association *p*-values for final viscosity (FV). The color and thickness of the bars are as previously described. The third track is the $–\log_{10}$ plot of the *p*-values of the *cis*-eQTL analysis done on the expression of the gene. The color and thickness of the bars are as previously described. The bottom track that shows SNP IDs with their alleles, the placement of the SNPs on the chromosome segment, and the haplotype blocks calculated using Gabriel's algorithm implemented in Haploview 4.2. (**b**)-(**c**) Represent the two alternative forms of the gene due to alternative splicing. The orientation of the strand indicates that this gene was annotated in the reverse strand.

Figure S10

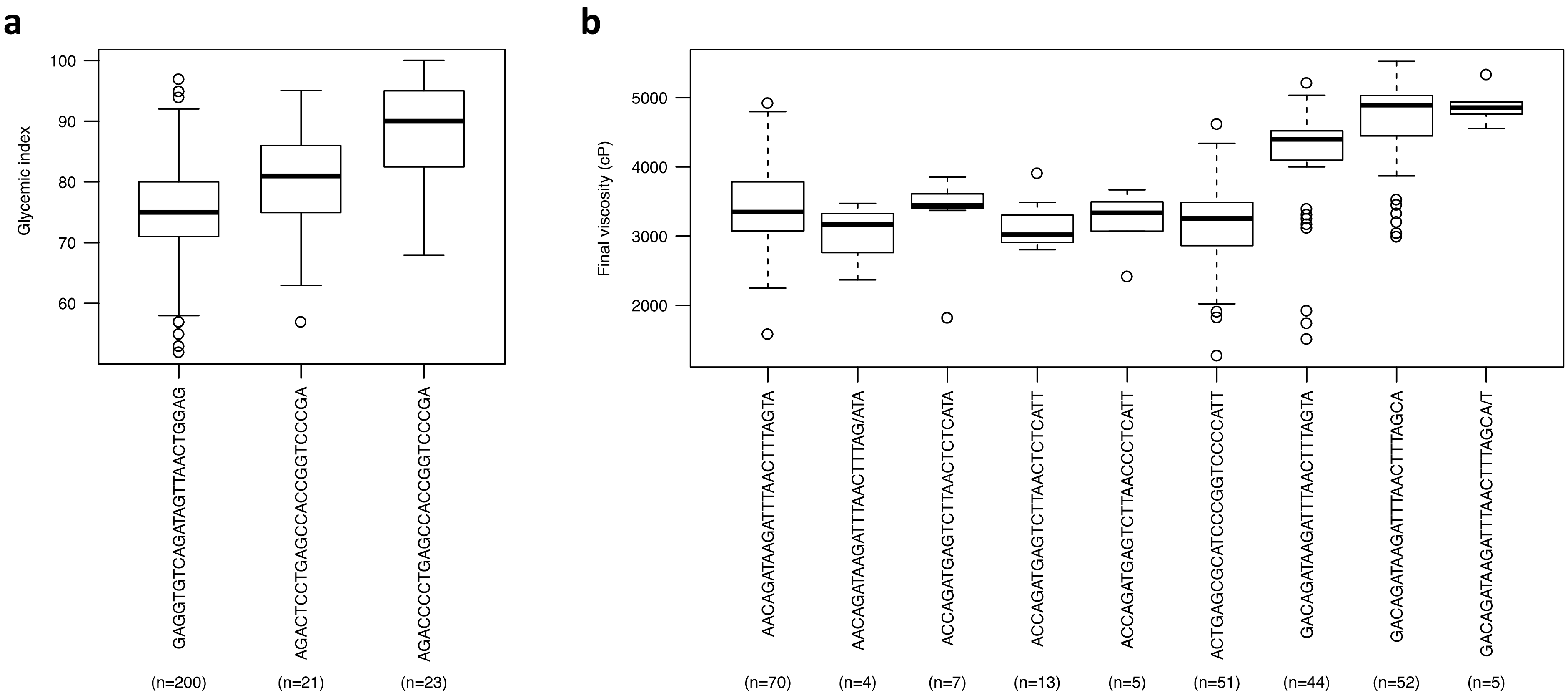

Figure S10 Phenotypic variation explained by haplotypes formed by significant SNPs in a gene encoding transmembrane glycosyl hydrolase (LOC_Os06g04169). (**a**) A total of 24 significant SNPs in the promoter (-1 kb) and genic regions of the gene (see **Figure S9a**) explaining phenotypic variation of glycemic index (GI). (**b**) Variation in final viscosity (FV) explained by the same SNPs (see **Figure S9a**).

Figure S11

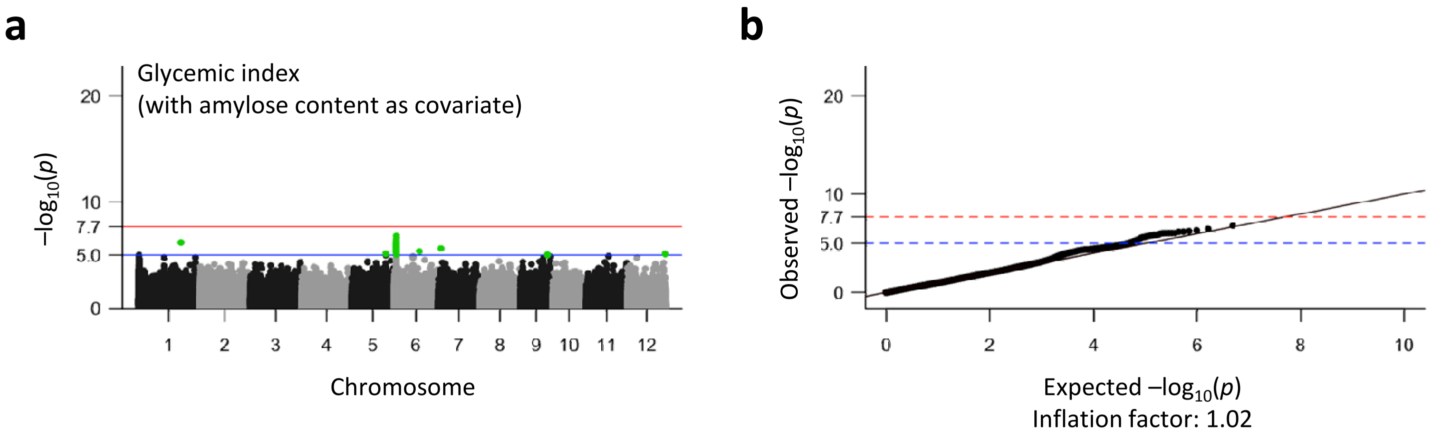

Figure S11 Genome-wide association study for glycemic index with amylose content as covariate. (**a**) Manhattan plot where the association significance threshold for the –log10 of the association *p*-values was at 7.7 after Bonferroni correction. The green dots indicate those SNPs with $p<1.0e\text{-}5$, which were within *q*-value significance (false discovery rate). (b) Quantile-quantile plot for the GWAS with inflation factor λ=1.02.

Figure S12

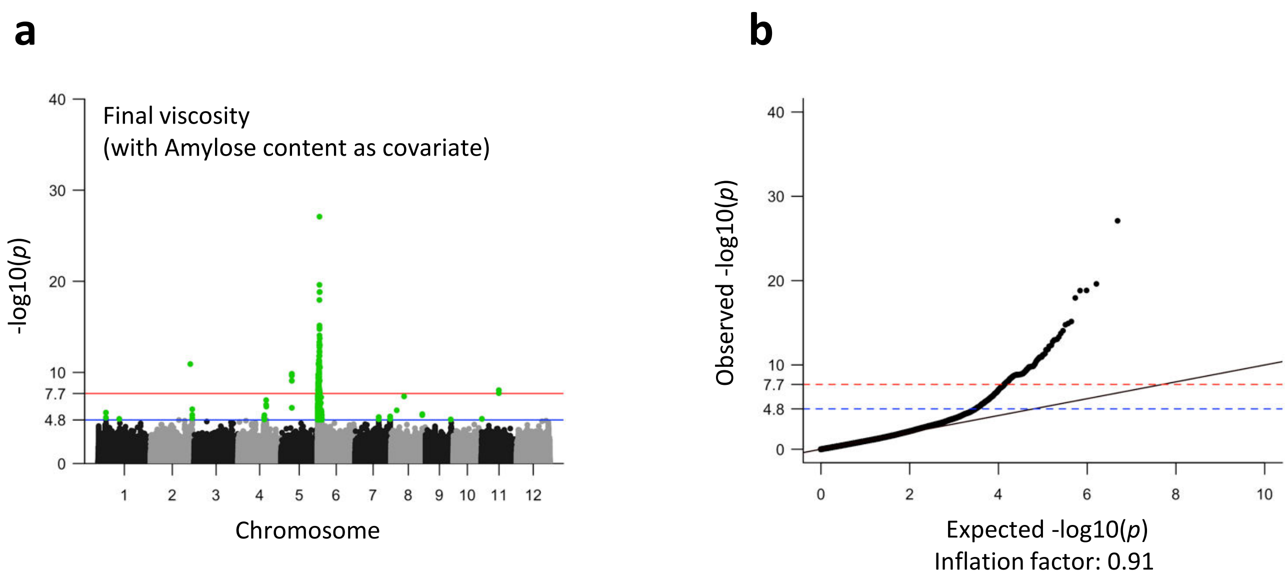

Figure S12 Genome-wide association study for final viscosity (FV) with amylose content (AC) as covariate. (**a**) Manhattan plot shows significant association signals in chromosomes 2, 5, 6, and 11 indicated by the red line at $-\log_{10}(p)=7.7$ that represents the Bonferroni-corrected *p*-value significance threshold. The blue line at $-\log_{10}(p)=4.8$ is the suggestive line represented by the association *p*-value that corresponds to the *q*-value<0.5 (false discovery rate). (**b**) Quantile-quantile plot where the inflation factor λ=0.91.

Figure S13

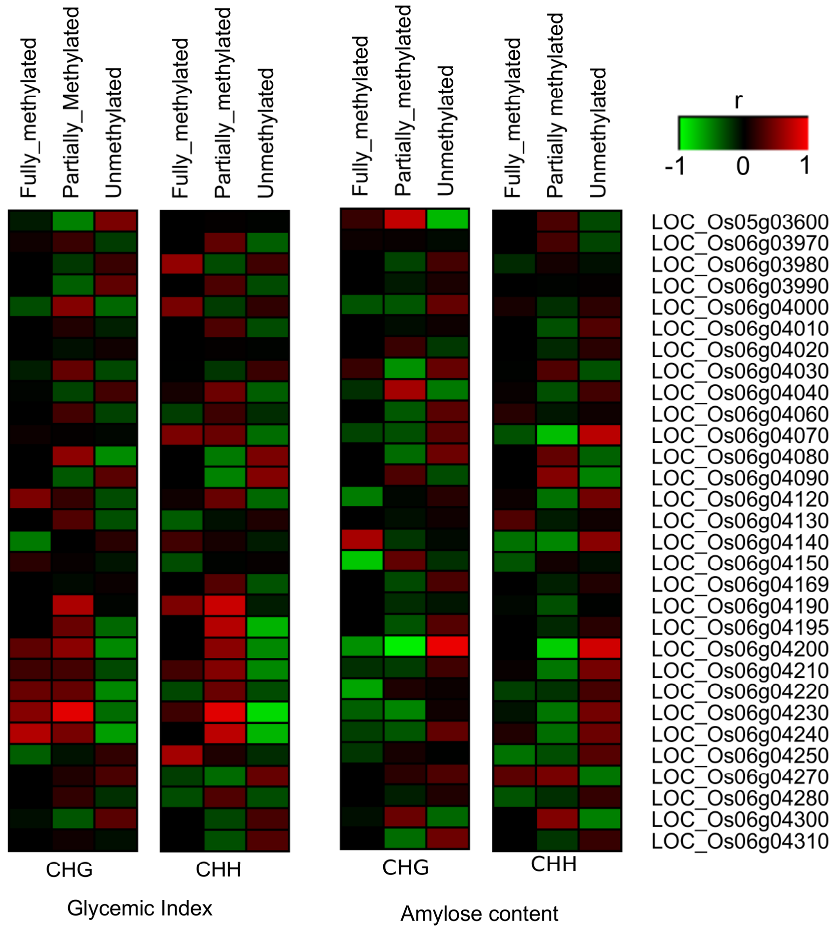

Figure S13: Correlation of GI and AC with the level of methylation existing in the genic region of the genes underlying *GI6.1* hotspot region, in the ten resequenced lines. **(a)** Effect of degree of methylation (CHH and CHG context) in the genic regions of the candidates in *GI6.1* region, on phenotype of GI and % amylose content in the ten resequenced lines. Fully methylated region depicted >90% methylated region, whereas, partially methylated region symbolizes the 10-90% of methylation in the region. Correlation coefficients ranged from -1 (green) to +1 (red).

Figure S14

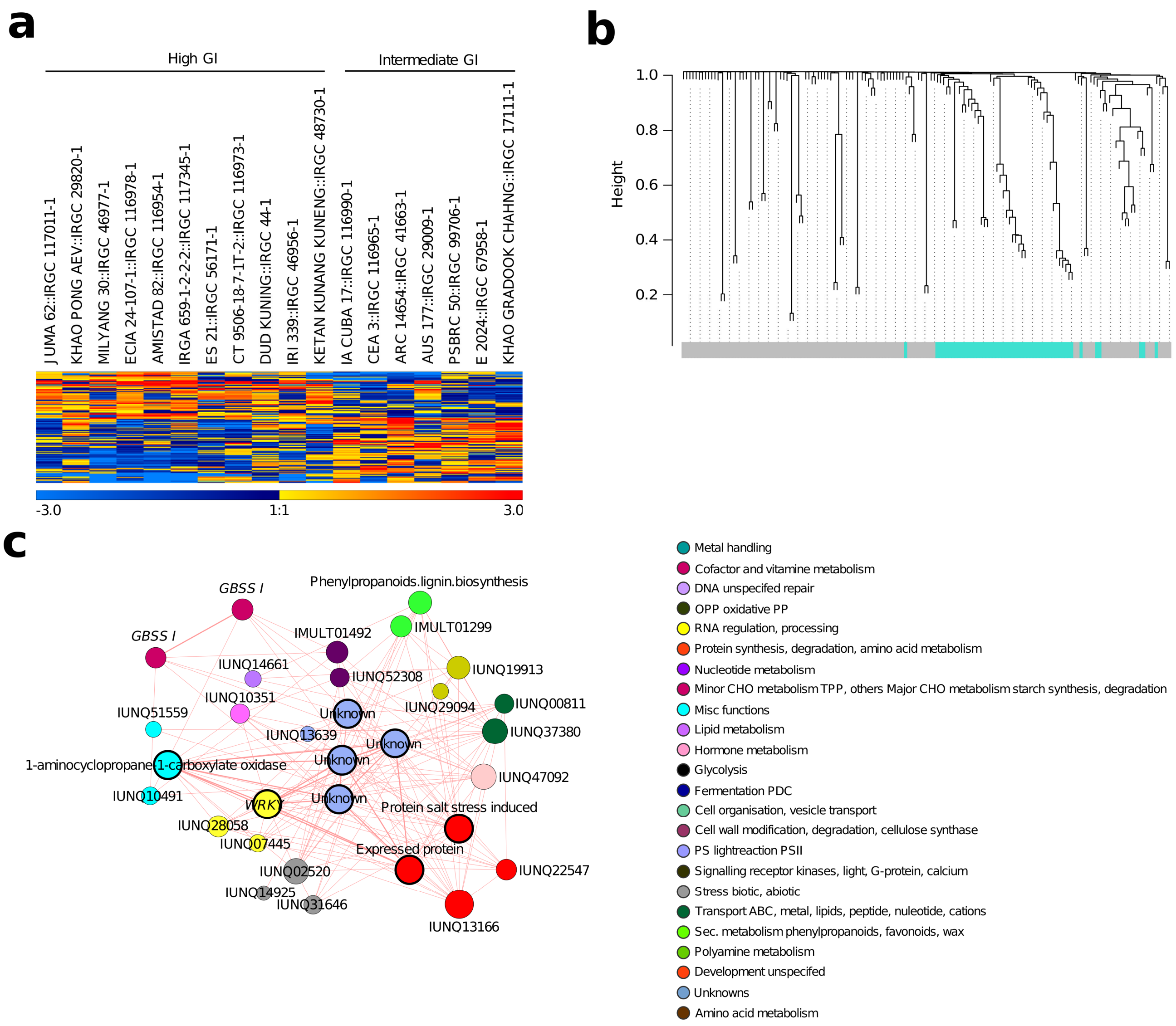
a
High GI
Intermediate GI
J UMA 62::IRGC 117011-1
KHAO PONG AEV::IRGC 29820-1
MILYANG 30::IRGC 46977-1
ECIA 24-107-1::IRGC 116978-1
AMISTAD 82::IRGC 116954-1
IRGA 659-1-2-2-2::IRGC 117345-1
ES 21::IRGC 56171-1
CT 9506-18-7-1T-2::IRGC 116973-1
DUD KUNING::IRGC 44-1
IRI 339::IRGC 46956-1
KETAN KUNANG KUNENG::IRGC 48730-1
IA CUBA 17::IRGC 116990-1
CEA 3::IRGC 116965-1
ARC 14654::IRGC 41663-1
AUS 177::IRGC 29009-1
PSBRC 50::IRGC 99706-1
E 2024::IRGC 67958-1
KHAO GRADOOK CHAHNG::IRGC 17111-1
-3.0
1:1
3.0
b
Height
1.0
0.8
0.6
0.4
0.2
c
Phenylpropanoids.lignin.biosynthesis
GBSS I
GBSS I
IMULT01492
IMULT01299
IUNQ14661
IUNQ19913
IUNQ52308
IUNQ10351
IUNQ29094
IUNQ00811
IUNQ51559
Unknown
IUNQ13639
IUNQ37380
Unknown
Unknown
1-aminocyclopropane-1-carboxylate oxidase
IUNQ47092
IUNQ10491
WRKY
Unknown
Protein salt stress induced
IUNQ28058
IUNQ07445
Expressed protein
IUNQ02520
IUNQ22547
IUNQ14925
IUNQ31646
IUNQ13166
Metal handling
Cofactor and vitamine metabolism
DNA unspecifed repair
OPP oxidative PP
RNA regulation, processing
Protein synthesis, degradation, amino acid metabolism
Nucleotide metabolism
Minor CHO metabolism TPP, others Major CHO metabolism starch synthesis, degradation
Misc functions
Lipid metabolism
Hormone metabolism
Glycolysis
Fermentation PDC
Cell organisation, vesicle transport
Cell wall modification, degradation, cellulose synthase
PS lightreaction PSII
Signalling receptor kinases, light, G-protein, calcium
Stress biotic, abiotic
Transport ABC, metal, lipids, peptide, nuleotide, cations
Sec. metabolism phenylpropanoids, favonoids, wax
Polyamine metabolism
Development unspecifed
Unknowns
Amino acid metabolism

Figure S14: Weighted gene co-expression analysis among high and intermediate GI lines. (a) heat map of differentially expressed genes between the high vs intermediate lines (Heat map scale ranges from min -3 to max +3 normalized expression values which are shown as blue to red color gradient representing the low and high expression, respectively). (b) Gene dendrogram with the turquoise clustered module in high vs intermediate. (c) Gene co-expression subnetwork of the turquoise module in high vs intermediate. Nodes in each network represent the gene and edges as the interaction, the variation of node size shows the different degree of connectivity and bordered wall nodes represents the hub. Functional categories based on the MapMan ontology term are shown in different colored nodes in the network.

Figure S15

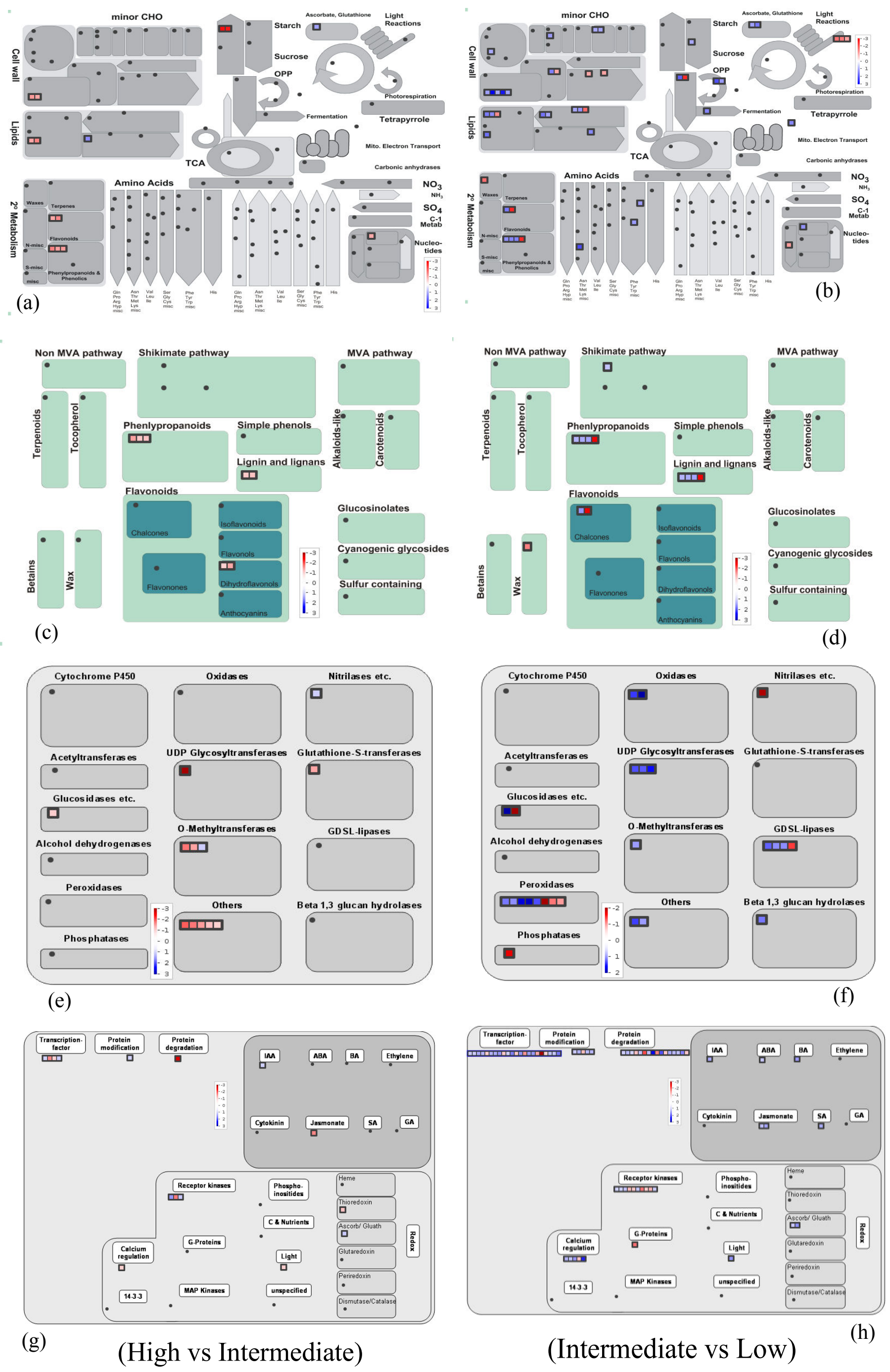

Figure S15 Overview of gene expression profile by MapMan. **(a, b)** metabolic response; **(c, d)** secondary metabolism; **(e, f)** enzyme families; **(g, h)** regulation overview by MapMan between high versus intermediate and intermediate versus low, respectively. (logFC±3, Blue color represents high expression, while red represents low).
