# Supplementary material for: Integrating a genome‐wide association study with a large‐scale transcriptome analysis to predict genetic regions influencing the glycaemic index and texture in rice
Source: Plant Biotechnol J. 2019 Jan 24;17(7):1261–75. doi: 10.1111/pbi.13051 (PMC6575982; doi:10.1111/pbi.13051)
Supplement: Supplementary file 3 — Appendix S1 Supporting experimental procedures. [file PBI-17-1261-s001.docx]

**Supporting experimental procedure: Appendix S1**

**Plant materials**

The plant materials used in this study included 305 *indica* varieties randomly selected from the 3,000 rice germplasm (The 3000 Rice Genomes Project, 2014) that are known to mature within 140 days. The rice was planted in three replicates using a random complete block design at the IRRI experimental field during the 2015 dry season at Zeigler Experimental station, International Rice Research Institute, Los Baños, Philippines. Each of these three replicates included four 12 m x 50 m blocks, and each block included 80.1 m x 4.2 m plots. Each plot included five rows with 24 hills per row. The hills were equally spaced at 20 cm x 20 cm to any of the four adjacent hills. The 305 varieties, including 15 filler varieties, were grouped into four, for a total of 80 per group. Each of these 80 varieties was randomly assigned to the 80 plots within each block, and each block was randomly placed within each replicate. The transplanting was performed 21 days after germination. The total N-P-K fertilizer used was 120-30-30, which was applied in three fractions of 30-30-30 at the basal stage, and the remaining 90 N were applied in equal fractions at the active tilling and panicle initiation stages. Other crop and field management were followed as per IRRI standard practices during harvesting, post-harvesting, and seed storage.

***In-vivo* GI evaluation**

Two milled rice accessions (GQ02497 and GQ02522) used in the study grown at the IRRI experimental station, Los Baños. The *in-vivo* GI of cooked rice samples was evaluated at the Food and Nutrition Research Institute (FNRI), Department of Science and Technology (DOST), Philippines. For cooking white rice, water to rice ratio was taken as 1.5:1 in the electric rice cooker. After cooking, rice was left undisturbed for 30 minutes before serving. A standard glucose drink (Medic Orange) was served as the reference food. In general, the control and test foods, contained equivalent of about 50 g of available carbohydrates were used for the test; although for GQ02522, 25% of medic orange was administered as a reference food and 25 g of available carbohydrate equivalent of the rice was fed due to inability of the subjects to consume the entire quantity within 15 minutes as required by protocol. Test foods were fed three times and the reference food twice, on separate days, after an overnight fast of 10-12 hours.

Twelve (12) apparently healthy adult humans (21-40 years old; 5 males, 7 females) were selected as the participants of the study upon meeting the following criteria: BMI of 18.5-22.9 kg/m2; fasting blood sugar (FBS) of 60-110 mg/dL; HbA1C<5.7%; non-smokers; without medication. Blood samples of approximately 0.3mL-0.4mL were collected through finger prick method before and after feeding the test/reference foods in microtainer tubes at 0, 15, 30, 45, 60, 90 and 120 minutes. The serum was separated from the blood using a refrigerated centrifuge and analyzed for glucose levels on the same day using a clinical chemistry analyzer. The area under the curve (AUC) for each food ignoring the area below the fasting level was calculated geometrically.

The glycemic index of the food was expressed as mean glucose response of the test food divided by the glucose response of the reference food following standard procedures established by Wolever et al. (Trinidad et al., 2013; Wolever et al., 1991).

Ethical clearance was given by the Institutional Human Ethics Committee of the FNRI-Department of Science and Technology (DOST), Philippines. The study participants were duly consented and signed the voluntary written consent forms.

**Texture and sensory evaluation**

Three low GI rice cultivars (GQ01795, GQ02522 and GQ02497) were used for texture and sensory evaluation. The paddy grains were first dried, dehulled and milled. A market sample of “Dinorado”, a popular rice variety of Philippines, was used as internal benchmark quality control. Texture profile analysis (TPA) was performed using three test portions (as replication); the grains in each replication were submerged in water inside a glass test tube and then sealed properly. Then, for cooking boiled in water bath (20 min) and placed in another water bath (50°C) to minimise retrogradation before conducting TPA. For each cooked test portion, three TPA replications were analyzed, and in each replicate, three intact cooked rice grains (not deformed) were evaluated in the texture analyzer.

For sensory evaluation, rice samples were cooked with equal ratio of water and then rice was mixed, keeping the rice adhering to sides and the bottom undisturbed. Then sub-samples were distributed into sealed glass cups before presenting for sensory evaluation. To minimise moisture loss, the rice cookers were quickly covered after subsamples were obtained. For keeping samples warm during the evaluation, subsamples were placed directly to the panelist’s cups, whenever requested. Panelists are generally trained before sensory descriptive profiling for the major fourteen attributes (Champagne et al., 2010). Panellists evaluated the intensity of each attribute following previously established 15-point reference scales (Goodwin et al., 1996). Rice tasting session occurred on two separate days to test the 3 low GI lines. However, Dinarado was used as internal control to monitor the reproducibility of panellist performance by following the standard rice tasting session protocols.

All statistical analyses were performed using R version 3.3.2 (released 2016, The R Foundation for Statistical Computing). The TPA and sensory evaluation followed a completely randomised experimental design.

**Phylogenetic analysis**

Only 636,974 SNPs without missing calls were included in the phylogenetic analysis. The DNA sequences using these SNPs were created for all varieties and saved in a FASTA file. This file was loaded in MEGA7 (Kumar et al., 2016) as non-protein coding DNA sequences, and a neighbour-joining tree was created using the Maximum Composite Likelihood nucleotide substitution model bootstrapped with 50 replications. All other parameters in MEGA7 were maintained at their default settings. The three varieties with the lowest GI values were highlighted.

**Linkage disequilibrium and haplotype analysis**

Two genotype data sets were prepared for the linkage disequilibrium (LD) analysis as follows: one data set was used to calculate the genome-wide LD decay, and the other data set was used to calculate the long-range LD decay within the region in which the association signal was detected. Prior to assembling these two SNP data sets, the source data were first filtered to retain only those SNPs with a minor allele frequency (MAF) of 5%.

The SNP data used to calculate the genome-wide LD decay contained markers that were systematically selected to correspond to 500-base-pair intervals on each chromosome. A sequential bidirectional scan was performed in cases where the i^th^ multiple of 500 base pairs landed on either a non-polymorphic site or a SNP position with a missing value. These genotype data had approximately equally spaced markers along each chromosome in the genome and an average ideal maximum resolution of four SNP markers for every two kilobases. Using the “--r2 dprime with-freqs” function in plink2, the r^2^ and D’ values of all pairs of SNPs within 500 kilobases were calculated. The “--ld-window” parameter was set to an unrealistically high number to ensure that all possible pairwise LD calculations for all SNPs within the prescribed window will be exhausted. The output data were then loaded in R, and the average r^2^ and D’ were calculated for each bin representing physical distances of 1 kb between any pair of SNPs. These average r^2^ and D’ values were then plotted against all 500 bins, and the point at which the LD breaks was indicated in the axes.

The other SNP data were used to visualize the LD decay within the region in which the association signals of both the GI and final viscosity (FV) were detected. All used SNPs within this region were pre-filtered to have an MAF ≥ 5%. The calculations of r^2^ and D’ were similarly performed in plink2, and the only difference was that the LD was calculated for SNPs with a maximum physical distance of 1,000 kb. The average r^2^ and D’ values were calculated and plotted similarly to the other data set.

Haplotype blocks were formed using Gabriel’s algorithm implemented in plink2 (Chang et al., 2015; Gabriel et al., 2002). The maximum block size was set at 200 kb, which is consistent with the consensus known maximum LD-decay span in *Oryza sativa* L. (Garris et al., 2003; Mather et al., 2007; McNally et al., 2009). No restrictions were set for the minimum block size in terms of the number of SNPs, and the 90% D’ confidence interval was set at 0.70-0.98, which is consistent with the D’ LD-decay value obtained in the LD-decay analysis. The other settings for the haplotype block estimation were maintained at their default values.

**Methylation analysis**

Ten developing rice grain samples at 16 DAF were selected from the rice diversity panel for the methylation. Total genomic DNA was extracted using the DNeasy Plant Mini Kit based on manufacturer’s protocols (Qiagen, Germany), and the total RNA was isolated as previously described (Butardo et al., 2017). Whole genome bisulphite sequencing analyses were performed by BGI Genomics. Whole genome bisulphite sequencing was performed after completing bisulphite treatment with the ZYMO EZ DNA Methylation-Gold Kit. Low-quality bases and adapters were removed using the NGStoolkit (http://www.nipgr.res.in/ngsqctoolkit.html). Each read with a length ≥70% and a ≥30 phred score was maintained for further analysis. We used Bismark (https://www.bioinformatics.babraham.ac.uk/projects/bismark/) to align the filtered WGBS (Whole genome bisulphite sequence) reads to the MSU7 version (Nipponbare) reference genome, which produced the corresponding SAM file for each sample. All default parameters were used for the alignment in Bismark. MethyleKit (https://www.ncbi.nlm.nih.gov/pmc/articles/PMC3491415/), which is an R package, was used to detect the levels of methylation at individual C bases for CG patterns. SAM file outputs from Bismark were used for the detailed analysis in MethyleKit. The methylation percentage in a promoter region of the identified genes was calculated after being divided into the following 3 categories: (1) fully methylated (where ≥90% of each base is methylated), (2) fully unmethylated (where ≤10% of each base is methylated) and (3) partially methylated (where the methylation level of each base is between 10% and 90%). The percentage of fully methylated, partially methylated and fully unmethylated regions was calculated based on the total C bases covered by a minimum of 5 reads and 20 phred score cut-off at the CG pattern. The correlation was calculated using the cor function in R based on the Pearson method between the methylation levels and amylose contents.

**Data for gene network analysis**

Accessions belongs to haplotypes based on their GI values (high, intermediate and low GI) were selected from the set of 200 *indica* lines. A microarray experiment was conducted strictly following the One-Color Microarray-Based Gene Expression Analysis protocol using a custom 8 × 60 K microarray slide for rice (Agilent, Germany). Briefly, cDNA synthesis and cRNA labeling were done using a single-color low input quick Amp labeling Kit. Microarray hybridization was conducted in a SureHyb chamber assembly using the large-volume Hi-RPM gene expression hybridization kit. Hybridization was done in a hybridization oven set at 60°C rotating at 10 rpm for exactly 17 h. Up to four microarray slides were processed and washed using the Gene Expression Wash buffer Kit with 0.005% Triton X-102. Microarray slides with an ozone barrier slide cover were read by a SureScan Microarray Scanner controlled by Scan Control software (Agilent Technologies) using a scan resolution of 3 µm double pass. Raw data were generated from the TIFF file using the Feature Extraction software (Agilent Technologies). The quality of the data was judged per batch using the PDF QC report and as a group using QC validation software. The data were normalized using GeneSpring GX (Agilent Technologies) following the quantile normalization algorithm.

**DEGs statistic**

The DEGs between high vs intermediate and intermediate vs low GI lines were calculated using the limma R package (Ritchie et al., 2015). This method follows an empirical Bayes method to shrink the probewise sample variances toward a common value and to augment the degrees of freedom for the individual variances. Bonferroni correction was used for P-values adjustment. The top-ranked genes were selected having an adjusted P-value below 0.05 and fold change above ±1.

**Co-expression Network analysis**

Weighted gene correlation network analysis WGCNA (Langfelder and Horvath, 2008) method in R was used to identify the clusters (modules) of densely connected correlated genes and derive the co-expression networks describing the pairwise relationships (Pearson) among gene transcript. The correlation matrix (coefficient =<0.75) was transformed into a matrix of connection strengths (an adjacency matrix) by raising the correlation matrix to the soft power β (beta) of 5 which was interpreted as soft threshold of the correlation matrix. Adjacency function α_ij_ = Power (s_ij_, β) ≡ |s_ij_| ^β^ where s_ij_ is the co-expression similarity, and a_ij_ represents the resulting adjacency that measures the connection strengths. The power β is chosen using the scale free topology criterion proposed by Zhang and Horvath (Zhang and Horvath, 2005). Following the TOM (topological overlap matrix) similarity algorithm for unsigned network the adjacency matrix is converted to TO (topological overlap) matrix. The topological overlap matrix (TOM) is given by Ω=[ωij]. ωij is a number between 0 and 1 and is symmetric (i.e, ωij= ωji). The rationale for using this similarity measure was to consider that nodes which are part of highly integrated modules, expected to have high topological overlap with their neighbors. Specifically the topological overlap matrix is given by

ωij

lij +aij

=

min{k_i_, k_j_}+1-a_ij_

l_ij_ = ∑α_iu_ α_uj_

where, denotes the number of nodes to which both i and j are connected, and ω indexes the nodes of the network. Genes were hierarchically clustered based on TO similarity. Modules with fewer than 30 genes were merged into their closest larger neighbor module. The visualization of co-expression network was done by using Cytoscape (Shannon et al., 2003).

**References**

Butardo, V.M., Anacleto, R., Parween, S., Samson, I., de Guzman, K., Alhambra, C.M., Misra, G. and Sreenivasulu, N. (2017) Systems Genetics Identifies a Novel Regulatory Domain of Amylose Synthesis. *Plant Physiol.* **173**, 887-906.

Champagne, E.T., Bett-Garber, K.L., Fitzgerald, M.A., Grimm, C.C., Lea, J., Ohtsubo, K.i., Jongdee, S., Xie, L., Bassinello, P., Resurreccion, A.P., Ahmad, R., Habibi, F. and Reinke, R.F. (2010) Important sensory properties differentiating premium rice varieties. *Rice* **3**, 270–281.

Chang, C.C., Chow, C.C., Tellier, L.C., Vattikuti, S., Purcell, S.M. and Lee, J.J. (2015) Second-generation PLINK: rising to the challenge of larger and richer datasets. *Gigascience* **4**, 7.

Gabriel, S.B., Schaffner, S.F., Nguyen, H., Moore, J.M., Roy, J., Blumenstiel, B., Higgins, J., DeFelice, M., Lochner, A. and Faggart, M. (2002) The structure of haplotype blocks in the human genome. *Science* **296**, 2225-2229.

Garris, A.J., McCouch, S.R. and Kresovich, S. (2003) Population structure and its effect on haplotype diversity and linkage disequilibrium surrounding the *xa5* locus of rice (*Oryza sativa* L.). *Genetics* **165**, 759–769.

Goodwin, H.L., Jr., Koop, L.A., Rister, M.E., Miller, R.K., Maca, J.V., Chambers, E., Hollingsworth, M., Bett, K.L., Webb, B.D. and McClung, A.M. (1996) Developing a common language for the U.S. rice industry: Linkages among breeders, producers, processors, and consumers. In: *TAMRC Consumer Product Market Research Report* p. 43. Texas Agricultural Market Research Center.

Kumar, S., Stecher, G. and Tamura, K. (2016) MEGA7: Molecular Evolutionary Genetics Analysis Version 7.0 for Bigger Datasets. *Molecular Biology and Evolution* **33**, 1870-1874.

Langfelder, P. and Horvath, S. (2008) WGCNA: an R package for weighted correlation network analysis. *BMC Bioinformatics* **9**, 559.

Mather, K.A., Caicedo, A.L., Polato, N.R., Olsen, K.M., McCouch, S. and Purugganan, M.D. (2007) The extent of linkage disequilibrium in rice (Oryza sativa L.). *Genetics* **177**, 2223-2232.

McNally, K.L., Childs, K.L., Bohnert, R., Davidson, R.M., Zhao, K., Ulat, V.J., Zeller, G., Clark, R.M., Hoen, D.R., Bureau, T.E., Stokowski, R., Ballinger, D.G., Frazer, K.A., Cox, D.R., Padhukasahasram, B., Bustamante, C.D., Weigel, D., Mackill, D.J., Bruskiewich, R.M., RÃ¤tsch, G., Buell, C.R., Leung, H. and Leach, J.E. (2009) Genomewide SNP variation reveals relationships among landraces and modern varieties of rice. *Proc. Natl. Acad. Sci. U. S. A.* **106**, 12273–12278.

Ritchie, M.E., Phipson, B., Wu, D., Hu, Y., Law, C.W., Shi, W. and Smyth, G.K. (2015) limma powers differential expression analyses for RNA-sequencing and microarray studies. *Nucleic Acids Res.* **43**, e47-e47.

Shannon, P., Markiel, A., Ozier, O., Baliga, N.S., Wang, J.T., Ramage, D., Amin, N., Schwikowski, B. and Ideker, T. (2003) Cytoscape: a software environment for integrated models of biomolecular interaction networks. *Genome Res.* **13**, 2498-2504.

The 3000 Rice Genomes Project (2014) The 3,000 rice genomes project. *Gigascience* **3**, 7.

Trinidad, T.P., Mallillin, A.C., Encabo, R.R., Sagum, R.S., Felix, A.D. and Juliano, B.O. (2013) The effect of apparent amylose content and dietary fibre on the glycemic response of different varieties of cooked milled and brown rice. *Int. J. Food Sci. Nutr.* **64**, 89-93.

Wolever, T.M., Jenkins, D.J., Jenkins, A.L. and Josse, R.G. (1991) The glycemic index: methodology and clinical implications. *The American journal of clinical nutrition* **54**, 846-854.

Zhang, B. and Horvath, S. (2005) A general framework for weighted gene co-expression network analysis. *Statistical applications in genetics and molecular biology* **4**.
